# Multidisciplinary Canadian consensus on the multimodal management of high-risk and radioactive iodine-refractory thyroid carcinoma

**DOI:** 10.3389/fonc.2024.1437360

**Published:** 2024-11-04

**Authors:** Shereen Ezzat, Jesse D. Pasternak, Murali Rajaraman, Omar Abdel-Rahman, Andrée Boucher, Nicole G. Chau, Shirley Chen, Sabrina Gill, Martin D. Hyrcza, Nathan Lamond, Marie-Hélène Massicotte, Eric Winquist, Ozgur Mete

**Affiliations:** ^1^ Endocrine Oncology Site Group, Princess Margaret Cancer Centre, Toronto, ON, Canada; ^2^ Department of Surgery, University Health Network – Toronto Western Hospital, Toronto, ON, Canada; ^3^ Department of Radiation Oncology, Dalhousie University, Halifax, NS, Canada; ^4^ Department of Oncology, University of Alberta, Edmonton, AB, Canada; ^5^ Endocrinology Division, Centre Hospitalier de l'Université de Montréal, Montréal, QC, Canada; ^6^ Division of Medical Oncology, British Columbia Cancer – Vancouver, Vancouver, BC, Canada; ^7^ Division of Endocrinology, Faculty of Medicine, University of British Columbia, Vancouver, BC, Canada; ^8^ Department of Pathology and Laboratory Medicine, Arnie Charbonneau Cancer Institute, University of Calgary, Calgary, AB, Canada; ^9^ Division of Medical Oncology, Nova Scotia Cancer Centre, Halifax, NS, Canada; ^10^ Division of Endocrinology, Department of Medicine, Centre Hospitalier Universitaire de Sherbrooke, Université de Sherbrooke, Sherbrooke, QC, Canada; ^11^ Verspeeten Family Cancer Centre at London Health Sciences Centre, London, ON, Canada; ^12^ Department of Pathology, Laboratory Medicine Program, University Health Network and University of Toronto, Toronto, ON, Canada

**Keywords:** thyroid cancer, targeted therapy, molecular diagnosis, radioiodine-refractory differentiated thyroid cancer, multidisciplinary

## Abstract

Most follicular cell-derived differentiated thyroid carcinomas are regarded as low-risk neoplasms prompting conservative therapeutic management. Here, we provide consensus recommendations reached by a multidisciplinary group of endocrinologists, medical oncologists, pathologists, radiation oncology specialists, a surgeon and a medication reimbursement specialist, addressing more challenging forms of this malignancy, focused on radioactive iodine (RAI)-resistant or -refractory differentiated thyroid carcinoma (RAIRTC). In this document we highlight clinical, radiographic, and molecular features providing the basis for these management plans. We distinguish differentiated thyroid cancers associated with more aggressive behavior from thyroid cancers manifesting as poorly differentiated and/or anaplastic carcinomas. Treatment algorithms based on risk-benefit assessments of different multimodal therapy approaches are also discussed. Given the scarcity of data supporting management of this rare yet aggressive disease entity, these consensus recommendations provide much needed guidance for multidisciplinary teams to optimally manage RAIRTC.

## 1 Introduction

Follicular cell-derived differentiated thyroid carcinomas (DTC), which include papillary thyroid carcinoma, follicular thyroid carcinoma, invasive encapsulated follicular variant papillary thyroid carcinoma, and oncocytic carcinoma of the thyroid, arise from genetically modified follicular cells in the thyroid gland. Therapy with ^131^I, or radioactive iodine (RAI), exploits follicular cells’ iodine uptake machinery to facilitate cytotoxicity. RAI is a mainstay of post-operative DTC treatment; however, there is a subset of patients (<5%) who develop RAI-resistant or -refractory differentiated thyroid carcinoma (RAIRTC) (1). RAIRTC typically develops due to change of functional differentiation status, which is frequently accompanied by loss of the sodium iodide symporter required for iodine uptake (1). There is also a subset of DTCs that exhibit high-grade pathological features (tumor necrosis and/or ≥5 mitoses per 2 mm^2^) with a clinical course similar to poorly differentiated thyroid carcinoma (PDTC) that can be frequently associated with RAI-refractory disease (2).

RAIRTC has a dismal prognosis among all follicular cell-derived differentiated thyroid cancer types, with a 10-year survival rate of only 10% (3). Considering the suboptimal therapeutic benefit of repeated RAI therapy in patients with RAIRTC, and the availability of effective treatment regimens such as the vascular endothelial growth factor receptor (VEGFR) tyrosine kinase inhibitors (TKi) (lenvatinib and sorafenib), early identification and prediction of RAIRTC is critical (4–6). Selective v-raf murine sarcoma viral oncogene homolog B1 (BRAF), rearranged during transfection (RET), and tropomyosin receptor kinase (TRK) inhibitors are also potentially available. Thus, molecular testing is an integral consideration in the clinical management of patients with RAIRTC (7). Ultimately, treatment decisions for these patients require management by a multidisciplinary team equipped to interpret diagnostic assessments and evaluate patient-specific factors (8).

A Canadian consensus statement on RAIRTC management was published in 2021, which focused on the multidisciplinary management of patients with the disease post-diagnosis (7). This statement, which involved active participation of nuclear medicine specialists, defined RAIRTC by outlining five key clinical scenarios indicative of disease: progression of thyroid cancer metastases despite RAI uptake; no RAI uptake in post-therapy scan despite known structural recurrent/metastatic disease; RAI uptake in some but not all cancer foci; thyroid cancer metastases progression despite cumulative RAI activity of >22.2 GBq (600 mCi); and no RAI uptake on diagnostic radioiodine scan (7).

Here, we aim to update and expand upon the previous statement by providing guidance on early identification of patients at risk of developing RAIRTC and practical referral and implementation strategies. This statement highlights the role of molecular testing for gaining prognostic and therapeutic insights and discusses multimodal options to optimize the management of RAIRTC.

## 2 Methods

### 2.1 Survey design and consensus development

A multidisciplinary committee of five Canadian physicians was assembled to lead development of the consensus recommendations: an endocrinologist, a radiation oncologist, an endocrine surgeon, an endocrine pathologist, and a medical oncologist. This committee met in November 2022 to identify the key topics related to identification and management of adult patients with RAIRTC in need of consensus, falling under three categories: diagnosis, therapeutics, and logistics/implementation.

**Figure 1:**
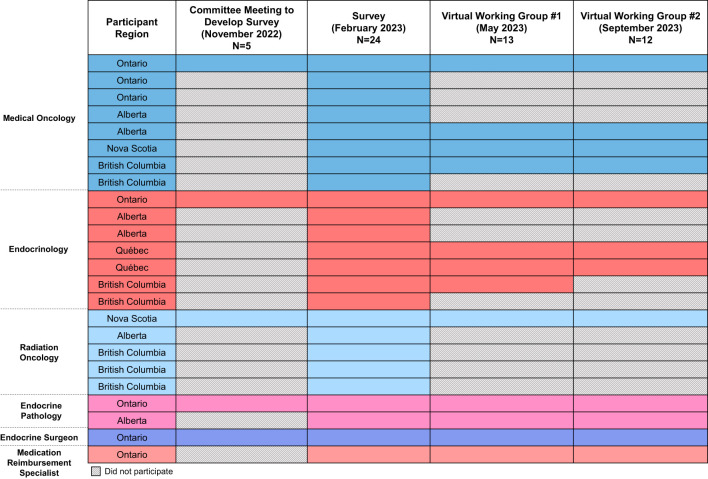
Overview of consensus participants. The specialties, regions, and extent of participation of consensus participants are summarized, with each row representing a different person. Participation in each step is indicated by a colored box, whereas absence/lack of participation is indicated by the hatched fill.

Following the committee meeting, a draft survey was developed and refined through asynchronous review by the committee. The survey was comprised of 31 questions (available in **Supplementary Material**), the majority of which were in multiple choice format with an optional open-ended response for rationale. All questions were optional to allow respondents of different specialties to only answer applicable questions as necessary. The survey was completed by 24 multidisciplinary participants across Canada, including the original committee, selected based on their expertise in their respective disciplines: seven from Ontario, seven from British Columbia, six from Alberta, two from Québec, and two from Nova Scotia (**Figure 1**).

Following survey completion, the results were compiled and grouped by topic. If ≥50% agreement (i.e., agree + strongly agree OR disagree + strongly disagree) was achieved on a survey question, a draft recommendation was developed. The committee and 7-8 survey respondents (**Figure 1**) then met virtually twice via working group meetings and provided asynchronous feedback on the draft recommendations, refining the recommendations as needed, until consensus was reached (i.e. ≥50% agreement). A consensus was unable to be reached on one draft recommendation, related to poly (ADP-ribose) polymerase inhibitor (PARPi) use, which was ultimately omitted from this document.

### 2.2 Literature search and evidence grading

A comprehensive literature search was conducted using PubMed (search strategy available in **Supplementary Material**) to determine the level of evidence supporting the consensus recommendations. The American College of Physicians’ (ACP) Grading System, as used by the 2015 American Thyroid Association Management Guidelines (9), was adopted for use in this consensus statement. We reviewed other appraisal systems but determined their complexity was not necessary given the low level of evidence available in this area. The quality of evidence for all recommendations was low or insufficient, based on the absence of randomized controlled trials/strong observational data inherent to this rare patient subpopulation. For topics where evidence was insufficient, recommendations were based on Expert Opinion and reflect physician experience as well as evidence from the management of other types of thyroid cancer. All recommendations are summarized in **Supplementary Table 1**, **Supplementary Material**.

## 3 Consensus recommendations

**Figure 2:**
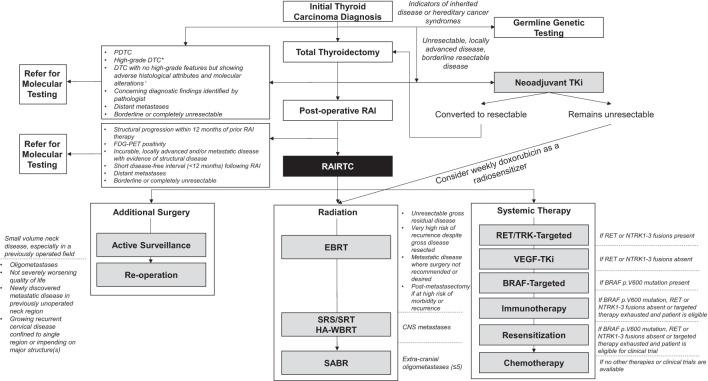
Recommended management options for high-risk and RAI-refractory follicular cell-derived differentiated thyroid cancer. All consensus recommendations are summarized in a management algorithm spanning from initial thyroid carcinoma diagnosis to development of RAIRTC. *Defined by the presence of tumor necrosis and/or mitotic count of at least 5 per 2 mm^2^ and no histologic or cytologic features of morphologic dedifferentiation (PDTC or ATC); ^†^Such as *TERT* promoter, *TP53* mutations, *PLEKHS1* promoter, miR21 overexpression, miR204 downregulation, DNA hypomethylation, chromosome 1q gain, as well as chromosome 5/7 duplication and near haploid genome, particularly in oncocytic carcinomas. ATC, anaplastic thyroid carcinoma; BRAF, v-raf murine sarcoma viral oncogene homolog B1; CNS, central nervous system; DTC, differentiated thyroid carcinoma; EBRT, external beam radiotherapy; FDG-PET, [18F]2-fluoro-2-deoxy-D-glucose-positron emission tomography; HA-WBRT, hippocampal-avoidance whole brain radiation therapy; miR, microribonucleic acid; NTRK, neurotrophic tyrosine receptor kinase; PDTC, poorly differentiated thyroid carcinoma; *PLEKHS1*, pleckstrin homology domain containing S1; RAI, radioactive iodine; RAIRTC, radioactive iodine-resistant differentiated thyroid carcinoma; RET, rearranged during transfection; SABR, stereotactic ablative radiotherapy; SRS/SRT, stereotactic radiosurgery or stereotactic radiotherapy; TERT, telomerase reverse transcriptase; TKi, tyrosine kinase inhibitor; TP53, tumor protein p53; TRK, tropomyosin receptor kinase; VEGF, vascular endothelial growth factor.

Consensus recommendations related to diagnosis, testing, and management flow for patients with DTC are outlined in **Figure 2**.

### 3.1 What features are suggestive of RAIRTC?

#### 3.1.1 Imaging features

While elevated serum thyroglobulin can be a marker for residual, recurrent, or metastatic disease in DTC, approximately one quarter of these patients have negative radioiodine whole-body scans (WBS) (10). Indeed, RAIRTC does not concentrate ^131^I and is therefore unable to be diagnosed/detected via radioiodine WBS. In contrast, FDG-PET scans, which visualize increased glucose metabolism found in tumors, have emerged as a valuable tool for the diagnosis and staging of RAIRTC. ^18^F-FDG uptake increases with the level of dedifferentiation and there is an inverse relationship between the ability to concentrate radioiodine and the uptake of ^18^F-FDG (10).

FDG-PET has shown sensitivity and specificity for the detection of recurrent and metastatic lesions of DTC in patients with signs of biochemical progression but negative iodine WBS (10–13). It is also capable of simultaneously detecting disease in both bone and soft tissues (10). Our group considers FDG-PET a complementary test, used on a case-by-case basis, for RAIRTC diagnosis and staging, with heterogeneity in terms of timing of when it should be used. While especially valuable in cases of discordance between structural imaging and clinical suspicion, access to FDG-PET scanning is variable across Canada, and thus it may not be feasible as part of routine monitoring paradigms. Indeed, discordance between biochemical parameters and structural imaging (e.g. rising thyroglobulin levels in the absence of anatomical disease measured by standard cross-sectional imaging) permits access to FDG-PET scanning in some Canadian provinces and is a valid scenario where this tool could be used (e.g. thyroid-stimulating hormone-stimulated FDG-PET). FDG-PET may also be valuable for staging of suspected RAIRTC resistant to treatment.

**Table:** 

Recommendation 3.1	Strength of Recommendation	Quality of Evidence
Follicular cell-derived non-anaplastic thyroid carcinoma at high risk of being RAIR can be identified by the presence of one or more of the following: • [18F]2-fluoro-2-deoxy-D- glucose-positron emission tomography (FDG-PET) positivity • PDTC • High-grade DTC (e.g., high- grade papillary thyroid carcinoma, high-grade follicular thyroid carcinoma, high-grade oncocytic carcinoma of the thyroid) • DTC with no high-grade features but showing adverse features (which may be histologic and/or molecular adverse [high-risk] features) strongly associated with RAIR disease	Weak	Low

#### 3.1.2 Histopathologic features

From a histopathological standpoint, PDTC represents a separate entity on the spectrum between DTC and anaplastic thyroid carcinoma (ATC), which is less likely to respond to RAI therapy (14). PDTC is defined as an invasive follicular cell-derived non-anaplastic thyroid carcinoma with solid/trabecular/insular growth that is unassociated with nuclear alterations of papillary thyroid carcinomas and that shows tumor necrosis and/or mitotic count of at least 3 mitoses per 2 mm^2^ (15). These tumors have intermediate behavior between DTC and ATC (15).

Similar to PDTCs, high-grade DTCs are also less likely to concentrate RAI (16). High-grade DTCs are defined by the presence of tumor necrosis and/or mitotic count of at least 5 per 2 mm^2^ and no histologic or cytologic features of morphologic dedifferentiation (PDTC or ATC) (15).

#### 3.1.3 Molecular features

Molecular alterations including telomerase reverse transcriptase *(TERT)* promoter, tumor protein p53 (*TP53)* mutations, pleckstrin homology domain containing S1 (*PLEKSH1)* promoter, microRNA (miR)21 overexpression, miR204 downregulation, DNA hypomethylation, chromosome 1q gain, as well as chromosome 5/7 duplication and near haploid genome, particularly in oncocytic carcinomas, have been recognized to be associated with disease progression (9, 15). Among these, *TERT* promoter alterations have shown a strong prediction for RAIRTC (17, 18).

Given the potentially poor outcomes associated with RAIRTC, it is of utmost importance to identify potential RAIRTC as early as possible to initiate appropriate referral and management paradigms. We acknowledge that true RAI refractoriness must ultimately be confirmed by attempting RAI therapy (and to qualify for systemic treatment); however, additional metabolic, histopathologic, genotypic, and molecular features can indicate the possibility of RAIRTC, prompting consideration of further investigation.

### 3.2 What types of patients should be referred for consideration of localized and/or systemic therapy?

We recommend a list of patient scenarios that should trigger referral for consideration of localized and/or systemic therapy. While those with structural disease progression despite RAI therapy are of highest priority, we also suggest scenarios that could be considered for referral, at the physician’s discretion. These scenarios, while less confirmatory of RAIRTC, are indicative of advanced disease warranting further investigation (15, 19). Although these recommendations may result in more patients being referred than usual, it will benefit patients to err on the side of caution and refer too soon rather than too late. We also note that patients with a high burden of disease and those at risk of complications should be fast-tracked for an expedited referral where possible.

The management of thyroid cancer in Canada, as well as globally, spans many disciplines, including primary care, medical oncology, general endocrinology, radiation oncology, nuclear medicine, head and neck surgery, otolaryngology surgery, and endocrine surgery. The physician responsible for care also varies depending on the stage of the patient journey. However, given the diversity of practitioners involved in care, our group felt it was essential to assign the responsibility of referral, so patients are adequately evaluated and directed appropriately.

**Table:** 

Recommendation 3.2a	Strength of Recommendation	Quality of Evidence
The following types of patients **should be referred** to a medical oncologist, endocrine oncologist, or discussed at a multidisciplinary tumor board: • Patients with structural progression within 12 months of prior RAI therapy The following types of patients **should be considered for referral** to a medical oncologist, endocrine oncologist, or discussed at a multidisciplinary tumor board: • Patients who are at high risk of RAIR follicular cell-derived non-anaplastic thyroid carcinoma (as defined above under section 3.1) • Patients who have incurable, locally advanced and/or metastatic disease with evidence of structural disease • Patients with short disease- free interval (<12 months) following RAI • Patients with FDG-PET avid disease • Patients with concerning histopathologic findings (as defined above under section 3.1)	Strong	Insufficient – Expert Opinion

**Table:** 

Recommendation 3.2b	Strength of Recommendation	Quality of Evidence
The following types of patients should be flagged for expedited referral: • Patients with rapidly progressing neck masses • Patients who are RAI-naïve or RAIR with symptomatic/ rapidly progressing disease (in high-risk population) • Patients whose disease is not amenable to local therapy and/ or already deemed inoperable or borderline resectable • Patients with high-grade follicular cell-derived non- anaplastic thyroid carcinoma (including PDTC and high- grade DTC) • Patients with bulky disease and/or of higher stage • Patients with disease at risk of causing morbidity or mortality, including but not limited to impending structural/ organ complications	Strong	Insufficient – Expert Opinion

**Table:** 

Recommendation 3.2c	Strength of Recommendation	Quality of Evidence
The clinician with thyroid cancer expertise who follows patients after RAI treatment should be the most responsible physician for identification and referral of patients with potential RAIRTC.	Strong	Insufficient – Expert Opinion

### 3.3 What is the role of germline (constitutional) genetic testing to predict prognosis?

Most thyroid cancers occur sporadically; however, familial non-medullary thyroid carcinoma occurs in ~3-9% of cases, 5% of which are syndromic (20). These hereditary predisposition syndromes for non-medullary thyroid cancer manifest with other types of lesions/tumors and include familial adenomatosis polyposis (FAP), *PTEN*-hamartoma tumor, Carney complex, Wermer syndrome (Multiple Endocrine Neoplasia Type 1 [MEN 1]), and *DICER1* syndrome (20, 21). Patients with syndromic thyroid cancer usually have known history of inherited predisposition syndrome or a family history of the associated manifestations. However, some syndromes, such as McCune-Albright, are not inherited (20). Histologic findings of the thyroid, such as multiple cellular follicular thyroid neoplasms (*PTEN*-hamartoma tumor syndromes) or multiple follicular adenomas with papillary architecture in association with multifocal follicular nodular disease and DTC (*DICER1* syndrome), should trigger the evaluation for an inherited predisposition syndrome.

A pre-operative diagnosis of most inherited predisposition syndromes does not generally alter the diagnostic approach for a thyroid nodule, with the exception of a known familial *RET* mutation (MEN2 syndrome), which may impact the extent of thyroidectomy or consideration for prophylactic thyroidectomy as well as guide management of related manifestations and monitoring of at-risk family members (20).

**Table:** 

Recommendation 3.3	Strength of Recommendation	Quality of Evidence
Genetic testing for disease-causing germline (constitutional) pathogenic variants (e.g., phosphatase and tensin homolog [*PTEN]*, *DICER1*, succinate dehydrogenase [*SDHx*], *TP53*) should be considered in the workup of select patients with diagnosed follicular cell-derived thyroid carcinoma, such as those with unique histomorphological and immunohistochemical features that may indicate inherited disease, or patients with hereditary cancer syndromes.	Weak	Low

### 3.4 What is the role of molecular (somatic) tissue testing?

After diagnosis, molecular tissue testing is typically not performed until patients have developed RAI-refractory disease. However, molecular testing can provide invaluable insights on prognosis and can identify patients with driver mutations eligible for efficacious and targeted therapies. Considering certain features previously identified, such as FDG-PET positivity, are indicative of RAIRTC, we recommend earlier use of molecular testing when such features are present in patients with potential RAIRTC to help optimize care.

**Table:** 

Recommendation 3.4a	Strength of Recommendation	Quality of Evidence
Molecular testing should be performed where clinically relevant and actionable, considering both therapeutic and potential prognostic implications.	Strong	Insufficient – Expert Opinion

**Table:** 

Recommendation 3.4b	Strength of Recommendation	Quality of Evidence
The following scenarios should trigger molecular testing: • Pre-operative: Triggered by the pathologist for patients with adverse histologic features (e.g., angioinvasive, high-grade features, morphologic dedifferentiation, adverse tumor subtypes) • Pre-operative: Triggered by the surgeon, radiation oncologist, or nuclear medicine physician for patients with distant metastases at diagnosis • Pre-operative: Triggered by the surgeon for patients with unresectable or borderline resectable disease who might be considered for systemic neoadjuvant therapy • Post-operative: Triggered by the radiation oncologist/ nuclear medicine physician at the first palliative (i.e., non-adjuvant) RAI treatment • Recurrence or progression: Triggered by the radiation oncologist or, rarely, nuclear medicine physician for patients with distant metastases at progression • Recurrence or progression: Triggered by the medical oncologist/endocrinologist (if not yet completed) when a patient is deemed inoperable • Recurrence or progression: Triggered by the surgeon when a patient is deemed borderline inoperable or completely inoperable	Strong	Insufficient – Expert Opinion

### 3.5 What advocacy regarding molecular testing is needed?

Molecular testing for biomarkers is broadly implemented in other areas of oncology, such as non-small cell lung cancer (NSCLC), even though the incidence of oncogenic driver alterations is not significantly higher than in DTC; *BRAF* p.V600 mutations occur in 3%, Kirsten rat sarcoma virus (*KRAS*) mutations in 20-30%, *RET* fusions in 1%, neurotrophic tyrosine receptor kinase (*NTRK*) fusions in <1%, and anaplastic lymphoma kinase (*ALK*) fusions in 3-5% of NSCLC (22). In comparison, *BRAF* p.V600E occurs in over 50% of adult papillary thyroid carcinoma (PTC) and *NRAS/HRAS/KRAS* mutations in 30-45% of follicular thyroid cancer and follicular variant PTC (23, 24). The *BRAF* p.V600E mutation is mutually exclusive with kinase fusions in the pre-treatment setting; thus in *BRAF* p.V600E-negative PTC, *RET* fusions occur in 14%, *NTRK* fusions in 8%, and *ALK* fusions in 3% of adult PTCs (25). A case of dual *NTRK* fusions in PTC has even been reported (26). Current access to molecular testing for thyroid cancer at tertiary centres in Canada is relatively limited in comparison with testing for NSCLC.

Molecular testing approaches for thyroid cancer are variable across Canada and globally, with differing selection of relevant tests and detection platforms. RNA or DNA next-generation sequencing (NGS) panels that detect alterations are preferred in patients with potential RAIRTC due to high sensitivity and maximal output of results for a given sample (i.e., detect multiple mutations/fusions) (19). This can be performed on core biopsy of the primary tumor, incisional/excisional biopsy of primary tumor or metastasis, or fine needle aspiration biopsy (FNAB) (27–30). We recommend patients with high-risk and RAIR follicular cell-derived non-anaplastic thyroid carcinoma have access to timely and high-quality molecular testing.

**Table:** 

Recommendation 3.5	Strength of Recommendation	Quality of Evidence
Canadian clinicians should advocate for improved molecular testing, including optimal timing, type of material used, and greater access at tertiary centres, to raise assessment of follicular cell-derived thyroid carcinoma to the level of other solid tumors.	Weak	Insufficient – Expert Opinion

### 3.6 What biomarkers should be tested?

*BRAF* mutations, *RET* fusions, and *NTRK1-3* fusions are essential to measure to determine eligibility for targeted therapies. *BRAF* p.V600E-specific immunohistochemistry has been found to be highly sensitive and specific for mutation detection (31–33), but variability in reproducibility/reliability in clinical practice is known to occur. *BRAF* p.V600E-specific immunohistochemistry is therefore recommended as a potential screening tool, if rigorously validated using molecularly characterized cases and available with rapid turnaround.

Additional biomarkers with potential prognostic implications are desirable to obtain, if accessible, to aid in clinical decision-making (34–37).

**Table:** 

Recommendation 3.6a	Strength of Recommendation	Quality of Evidence
The following biomarkers are **essential** to obtain in patients with RAIRTC: • *BRAF* p.V600E-specific immunohistochemistry • *BRAF* molecular • *RET* fusion • *NTRK* fusions (*NTRK1*, *NTRK2*, *NTRK3*)	Strong	Insufficient – Expert Opinion

**Table:** 

Recommendation 3.6b	Strength of Recommendation	Quality of Evidence
The following biomarkers are **desirable** to obtain in patients with RAIRTC if possible, considering sample availability and testing accessibility: • *NRAS* • *HRAS* • *KRAS* • *ALK* fusion • Peroxisome proliferator activated receptor gamma (*PPARG*) fusion • *ALK* fusion-specific immunohistochemistry • *TERT* promoter alterations • NUT midline carcinoma family member 1 (*NUTM1*) • PTEN immunohistochemistry • Succinate dehydrogenase complex iron sulfur subunit B (SDHB) immunohistochemistry • Pan-RAS Q61R mutation- specific immunohistochemistry • 5-hydroxymethylcytosine (5- hmC) immunohistochemistry	Weak	Insufficient – Expert Opinion

### 3.7 What is the role of re-operation?

Repeat resections in patients with potential RAIRTC must be approached cautiously, as re-operative thyroid surgery has been shown to have high rates of post-operative morbidity, including both transient (7.1%) and permanent (2.7%) hypoparathyroidism, and iatrogenic unilateral recurrent laryngeal nerve (RLN) palsy (1.6%), specifically in scenarios where the nerve is functioning pre-operatively (38).

Active surveillance may be considered, instead of re-operation, in those with small volume neck disease in a previously operated field. These patients should not have progressively enlarging metastatic lymph nodes or aggressive cytological features (9). Active surveillance requires informed surgical discussion, patient compliance, and an experienced multidisciplinary team with high-quality monitoring tools (20).

Patients who should be considered for re-operation include those with oligometastatic, rapidly progressive or symptomatic disease, newly discovered metastatic disease in the neck or where recurrent disease is considered to potentially threaten major structures (39). Re-operation may also be considered based on patient/endocrinologist preference, where it would be tolerated by the patient (20). Radiation or local therapies may be an alternative to surgery for inoperable patients (see sections 3.9-3.10).

Eligibility for resection should consider the impact on patient quality of life in addition to technical feasibility of the surgery. Indeed, surgical removal of disease invading the trachea, esophagus, or larynx may be particularly detrimental to patients’ quality of life by impacting their airway, speech, and swallowing (40). Patients who are unresectable or borderline resectable are considered for alternative treatments; however, the definition of “borderline resectable” disease is variable across surgeons. We recommend borderline resectable thyroid cancer be defined as: large volume cervical disease, which would preclude likely R0 resection, including invasion into critical structures such as larynx, major vascular structures, or large segment of trachea.

While the notion of borderline resectable thyroid cancer has not been discussed at length in the literature given its rarity in this population, other similar progressive cancers have been studied at length when scenarios such as this are encountered. Certain cancers, such as pancreatic and other solid organ malignancies, are similarly progressive and fatal to advanced stages of undifferentiated, RAIR, and anaplastic cancer, and have been shown to have dismal operative outcomes (41–44). Innovative strategies such as neoadjuvant targeted or chemotherapy can create a hope for positive outcome from subsequent surgical management. Surgical oncological principles such as these should be applied to both classifying borderline resectable thyroid cancer as well as determining treatment strategies to yield better outcomes for these patients.

**Table:** 

Recommendation 3.7a	Strength of Recommendation	Quality of Evidence
Active surveillance is recommended in patients with small volume neck disease, especially in a previously operated field.	Weak	Low

**Table:** 

Recommendation 3.7b	Strength of Recommendation	Quality of Evidence
Resection of recurrent/metastatic disease should be considered in the following scenarios: • Patients with oligometastases • Patients in whom it would not severely worsen quality of life • Patients with newly discovered metastatic disease in the neck in areas without previous operation • Patients with growing recurrent cervical disease confined to a single region or close to major structure with impending invasion	Weak	Insufficient – Expert Opinion

**Table:** 

Recommendation 3.7c	Strength of Recommendation	Quality of Evidence
Borderline resectable follicular cell-derived thyroid carcinoma should be defined as large volume cervical disease, which would preclude likely R0 resection due to either bulky and/or widespread lymphadenopathy (e.g., level VII or retropharyngeal) and/or invasion into critical structures such as larynx, major vascular structures, or large segment of trachea.	Weak	Insufficient – Expert Opinion

### 3.8 How should patient airway be managed?

In the absence of data on airway management in DTC, we use evidence in ATC as a guide. Tracheostomy may be offered as a palliative approach to provide symptom relief. Indeed, mortality due to airway compromise occurs in up to 60% of patients (45). However, upper airway obstruction is often present despite tracheostomy, and the intervention is associated with risk of major hemorrhage and decreased quality of life (e.g., tumor can erode the tracheostomy site) (45–47). It is therefore recommended to avoid tracheostomy for as long as possible because of the potential complications and deterioration of quality of life. Alternatively, once a patient develops acute symptoms, such as stridor or unmanageable secretions, a tracheostomy may be considered (45). Indeed, complete resection of disease without the need for tracheostomy has been reported with use of neoadjuvant targeted therapy for ATC (46).

Tracheal fistulization following TKi therapy has been reported in rare instances (48–50). Despite this, even in cases with higher rates of fistulization/perforation, disease control and continued survival were observed (51). Furthermore, while tumor infiltration and histological type may be risk factors for fistulization, decreasing the TKi dose did not impact fistula risk (51). Therefore, given these observations, we recommend not delaying TKi due to the concern of rare risks of tracheal fistulization. Thyroid surgery specialists should review the extent of disease, including transmural invasion into trachea and esophagus simultaneously as highest risk features for trachea-esophageal fistula to occur on use of TKi.

**Table:** 

Recommendation 3.8a	Strength of Recommendation	Quality of Evidence
When deciding about airway management in patients with locally advanced and/or progressive unresectable or borderline resectable disease, prior to institution of systemic TKi therapy, patient quality of life and end-of-life wishes should be considered before tracheostomy.	Weak	Insufficient – Expert Opinion

**Table:** 

Recommendation 3.8b	Strength of Recommendation	Quality of Evidence
TKi may be considered prior to tracheostomy in select patients with careful consideration of risk versus benefit and in discussion with the patient.	Weak	Insufficient – Expert Opinion

**Table:** 

Recommendation 3.8c	Strength of Recommendation	Quality of Evidence
Multikinase inhibitor treatment should not be delayed in select cases due to perceived risk of complications, including tracheal fistulization.	Weak	Insufficient – Expert Opinion

### 3.9 What is the role of radiotherapy?

Published studies of EBRT for DTC do not support improved overall survival or rates of distant metastases (52). However, there is evidence that EBRT improves locoregional control with acceptable toxicity, especially with use of modern precision radiation therapy technologies (19, 52–54). Consistent with published guidelines, we recommend EBRT in select cases for locoregional control (7, 9, 55, 56). Weekly doxorubicin may also be considered to help sensitize to radiation (57).

Consistent with published guidelines, we recommend SRS/SRT be offered to eligible patients with limited central nervous system metastases after appropriate neurosurgical consultation (9, 55). The treatment approach (i.e., use of SRS, SRT, and/or hippocampal-avoidance whole brain radiation therapy [HA-WBRT]) should be decided based on the extent and number of central nervous system metastases present.

Consistent with published guidelines, we recommend SABR for treatment of oligometastases (extra-cranial, bony, or soft-tissue) (55). There is no consensus on the precise definition of the oligometastatic state or clarity on how many metastatic lesions are amenable to ablative therapies that may benefit the patient. Although the definition of oligometastatic disease varies from 3-5 metastatic lesions in clinical trials (58) and studies with up to 10 metastases or more are ongoing (59, 60), Phase II studies show favorable progression-free survival and local control were observed after SABR in select patients with up to 5 metastases (61, 62). Despite the development of thyroid cancer hematogenous metastases, disease progression is relatively indolent with a generally longer survival than in those with similar advanced disease due to other primary malignancies. Therefore, aggressive management of patients who progress to M1 thyroid cancer, including those with high-risk or RAIRTC, is indicated, especially in those who are younger or have a good performance status (63).

**Table:** 

Recommendation 3.9a	Strength of Recommendation	Quality of Evidence
External beam radiotherapy (EBRT) should be considered in patients who have unresectable gross residual disease, very high risk of recurrence in neck despite all gross disease resected, metastatic disease where surgery is not recommended or desired, or post-metastasectomy if risk or morbidity of recurrence remains high (e.g., brain metastases resection, spine metastases resection).	Weak	Low

**Table:** 

Recommendation 3.9b	Strength of Recommendation	Quality of Evidence
Stereotactic radiosurgery or stereotactic radiotherapy (SRS/SRT) should be offered to eligible patients with central nervous system metastases after appropriate neurosurgical consultation.	Weak	Low

**Table:** 

Recommendation 3.9c	Strength of Recommendation	Quality of Evidence
Stereotactic ablative radiotherapy (SABR) for extra-cranial metastases should be considered for selected patients with ≤5 oligometastases.	Weak	Low

### 3.10 What is the role of alternative locoregional treatments?

Alternative treatments such as ethanol or radiofrequency ablation may be considered for locoregional control of lymph node metastases, as a directed approach for progressive/symptomatic disease (7, 9, 55). For example, a growing symptomatic lymph node in the lateral neck could be targeted with ablative therapy.

**Table:** 

Recommendation 3.10	Strength of Recommendation	Quality of Evidence
Alternative locoregional treatments such as ethanol or radiofrequency ablation may be considered in patients with growing cervical metastatic disease in previously operated fields, safely away from critical structures.	Weak	Low

### 3.11 What is the role of neoadjuvant TKi?

Unresectable DTC occurs in <10% of advanced DTC (64). Patients with unresectable DTC have poor outcomes, with a 5-year cumulative survival rate of 21.5% seen in a retrospective study of 22 patients (64). These patients are also typically unable to qualify for clinical trials as the lack of thyroidectomy means RAI cannot be attempted, and thus RAI refractoriness cannot be proven. In many other disease sites, including rectal cancer and esophagogastric cancer, neoadjuvant therapy prior to surgical resection has been standard of care for decades (65, 66). TKis have recently been reported to have a role in neoadjuvant treatment of unresectable or locally advanced DTC to reduce tumor volume and surgical morbidity (67–73). This has also been observed in ATC and medullary thyroid cancer (46, 74, 75). The 2023 National Comprehensive Cancer Network (NCCN) guidelines also recommend systemic therapy be considered for tumors that are not surgically resectable, or enrollment in neoadjuvant clinical trials, of which there are multiple ongoing (NCT04321954, NCT04180007, NCT04524884) (55).

**Table:** 

Recommendation 3.11	Strength of Recommendation	Quality of Evidence
Neoadjuvant TKi should be considered for those with unresectable and borderline resectable locally advanced thyroid carcinoma who may not have received RAI.	Strong	Low

### 3.12 What is the role of targeted therapy?

Genotype-directed targeted therapies currently available in Canada include dabrafenib (+/- trametinib)/vemurafenib (*BRAF* p. V600E mutation; off-label for DTC), selpercatinib (*RET* fusions), and larotrectinib/entrectinib (*NTRK* fusions). While VEGFR-targeting multikinase inhibitors lenvatinib, sorafenib, and cabozantinib are currently indicated for systemic treatment of RAIRTC, they can be associated with considerable adverse effects. In the SELECT trial of lenvatinib, ~76% of patients experienced grade 3 or higher treatment-related adverse events, with 14.2% of patients discontinuing the study drug due to adverse events compared to 2.3% with placebo (3). The most common adverse effects associated with lenvatinib were hypertension, diarrhea, and fatigue/asthenia (3). Although the populations are small, due to the rarity of the driver mutations being targeted, and have not been compared head-to-head, genotype-directed targeted therapies show high response rates and comparably lower serious adverse events compared to lenvatinib (**Table 1**).

**Table 1:** Efficacy and safety of targeted precision therapeutics in non-medullary thyroid carcinoma.

Treatment	Mechanism of Action (109)	Response	Grade ≥3 Treatment-related Adverse Events
Lenvatinib (n=261) (3)	VEGFR, PDGFR, EGFR, *RET*, KIT	Response rate* – 64.8%	76%
Dabrafenib (n=26) (110)	*BRAF* p.V600E	Objective response rate^†^ – 42%	58%
Dabrafenib + trametinib (n=27) (110)	Dabrafenib: *BRAF* p.V600E Trametinib: MEK1, MEK2	Objective response rate^†^ – 48%	48%
Vemurafenib, no prior VEGFR TKi (n=26) (111)	*BRAF* p.V600E	Best overall response^‡^ (PTC) – 38.5% (0% CR)	65%
Vemurafenib, prior VEGFR TKi (n=22) (111)	*BRAF* p.V600E	Best overall response^‡^ (PTC) – 27.3% (0% CR)	68%
Selpercatinib (n=19) (112)	*RET*	Objective response^II^ (non-MTC) – 58%	30% (n=162, includes MTC)
Larotrectinib – Pooled (n=21) (113)	TRKi: TRKA, TRKB, TRKC	Objective response rate^¶^ (DTC) – 86%	7% (n=21, pooled thyroid population)
Entrectinib (n=13) (114)	TRKi: TRKA, TRKB, TRKC *ALK*, ROS1	Objective response rate** (thyroid cancer) – 53.8%	38.9% (n=193, *NTRK* fusion population)

*Defined as the best objective response (complete or partial) according to RECIST 1.1.

^†^Defined as the proportion of patients who had a CR, PR, or MR within the first six cycles. CR and PR were defined by RECIST 1.1, and MR was defined as 20-29% decrease in the sum of diameters of target lesions compared to baseline.

^‡^Defined as the proportion of patients with a CR or PR, according to RECIST 1.1, as assessed by the investigator.

^II^Defined as CR or PR, investigator assessment, according to RECIST 1.1.

^¶^Defined as the proportion of patients with confirmed CR or PR as best overall response, assessed by the investigator according to RECIST 1.1.

**Defined as the proportion of patients with confirmed CR or PR as best overall response, by BICR.

ALK, anaplastic lymphoma kinase; BICR, blinded independent central review; BRAF, v-raf murine sarcoma viral oncogene homolog B1; CR, complete response; DTC, differentiated thyroid carcinoma; EGFR, epidermal growth factor receptor; MR, minor response; MTC, medullary thyroid cancer; NTRK, neurotrophic tyrosine receptor kinase; PDGFR, platelet-derived growth factor receptor; PR, partial response; PTC, papillary thyroid carcinoma; RECIST, Response Evaluation Criteria in Solid Tumors; RET, rearranged during transfection; TKi, tyrosine kinase inhibitor; TRKi, tropomyosin receptor kinase inhibitor; VEGFR, vascular endothelial growth factor receptor.

In the absence of formal head-to-head comparisons but given the favorable efficacy/safety profile of targeted therapies, we recommend patients with confirmed, clinically actionable genomic alterations be considered for targeted therapy. While the response rates for *NTRK* and *RET* fusion-targeting therapies appear to be promising, supporting their use before lenvatinib in eligible patients, we would not recommend routine use of BRAF inhibitors before lenvatinib, given their lower efficacy and weaker evidence.

**Table:** 

Recommendation 3.12	Strength of Recommendation	Quality of Evidence
Patients with confirmed, clinically actionable genomic alterations should be considered for targeted therapy, considering individual efficacy/safety needs and access.	Strong	Low

### 3.13 What is the role of chemotherapy/immunotherapy?

Immune checkpoint inhibitors, including antibodies against cytotoxic T-lymphocyte associated protein 4 (CTLA-4) and programmed cell death protein 1 (PD-1), have shown promise in cancer types such as melanoma, NSCLC, and head and neck cancers (76–79). Indeed, tumoral programmed cell death-ligand 1 (PD-L1) expression has been observed in thyroid carcinomas (80, 81) and has been associated with increased risk of recurrence and poor prognosis (81, 82). In the Phase 2 KEYNOTE-158 study, pembrolizumab was found to be effective (~7% overall response rate) for a small subset of patients with advanced DTC, regardless of tumor PD-L1 status, with manageable toxicities (83). Responses to other immunotherapies have also been reported in DTC (84, 85).

Despite these preliminary data, the use of immunotherapy/immune checkpoint inhibitors in thyroid cancer is still new. Thus, we recommend immune checkpoint inhibitors if no other treatments are available and patients are eligible (e.g. DNA mismatch repair deficient).

Chemotherapy (i.e., doxorubicin alone and in combination with other cytotoxic therapy, such as cisplatin) for patients with RAIRTC is generally considered ineffective, with response rates of ~20% (56, 86, 87); however, data are limited and large trials in contemporary thyroid cancer populations have not yet been conducted. Case studies have shown unique success of chemotherapy (88–92). Given the limited evidence, generally low response rates, and risk of adverse events, chemotherapy should be considered as a last resort, consistent with treatment guidelines (9, 56).

**Table:** 

Recommendation 3.13a	Strength of Recommendation	Quality of Evidence
In patients for whom other modalities and therapeutics have been exhausted, who do not have actionable targets, and are eligible, immune checkpoint inhibitors could be considered as treatment.	Weak	Low

**Table:** 

Recommendation 3.13b	Strength of Recommendation	Quality of Evidence
Although evidence is very limited, chemotherapy may be considered in select cases where there are no other therapeutic options, including targeted treatment, immune checkpoint inhibitors, or clinical trials/research protocols.	Weak	Low

### 3.14 What is the role of RAI resensitization?

Efforts have been made to resensitize advanced thyroid tumors to RAI by inducing redifferentiation and/or restoring uptake of iodine. Retinoic acids, histone deacetylase (HDAC) inhibitors, sorafenib, and PPARγ agonist rosiglitazone have been investigated, but with limited success (93–98). Larotrectinib was also observed to re-induce RAI uptake in *NTRK* rearranged PTC (99). Loss of the sodium iodide symporter, NIS, has been shown to occur when *BRAF* p.V600E is present (100, 101). Thus, the most promising resensitizing therapies are those that act on *BRAF*: BRAF inhibitor dabrafenib and downstream MEK inhibitors trametinib and selumetinib. While data have shown increased radioiodine avidity/uptake post treatment with BRAF/MEK inhibitors (102–105), re-induction of RAI uptake is variable, with co-occurrence of *TERT* mutations with *NTRK* fusions as a possible contributor (106 107). Additionally, a recent Phase 3 trial showed the addition of selumetinib to adjuvant RAI did not significantly improve 18-month complete remission (CR) rate versus placebo plus RAI in patients with DTC at high risk of primary treatment failure (108). Given the limited evidence and disappointing results of the selumetinib Phase 3 trial, we recommend resensitization only be attempted as part of a clinical trial, with careful monitoring.

**Table:** 

Recommendation 3.14	Strength of Recommendation	Quality of Evidence
RAI resensitization therapy should ideally be considered as part of a clinical trial.	Weak	Low

## 4 Conclusion

Thyroid cancer management can be relatively straightforward for the large proportion of patients diagnosed with well differentiated disease. This makes the recognition of the much less frequent but problematic cases more challenging. With this perspective in mind, we provide the evidence underlying clinical, radiographic, histomorphologic, and molecular hallmarks that portend more aggressive disease behavior. Tailoring a management strategy that optimizes risks versus benefits requires a thoughtful multidisciplinary approach. This includes multimodal therapies that consider the immediate and longer-term objectives for each patient. The hope is that such management paradigms will offer strategic pathways that can evolve as advances in their respective disciplines are achieved.
