# Supplementary material for: Multidisciplinary Canadian consensus on the multimodal management of high-risk and radioactive iodine-refractory thyroid carcinoma
Source: Front Oncol. 2024 Nov 4;14:1437360. doi: 10.3389/fonc.2024.1437360 (PMC11570806; doi:10.3389/fonc.2024.1437360)
Supplement: Supplementary file 1 [file DataSheet1.docx]

Supplementary Material

# Supplementary Data

**Survey Questions**

1. What is your specialty?
   1. Endocrinology
   2. Pathology
   3. Medical Oncology
   4. Surgery
   5. Radiation Oncology
   6. Nuclear Medicine
   7. Other: [open response]

**Diagnosis**

*Nomenclature*

1. The consensus paper should use the term radioactive iodine-refractory/resistant (RAI-R) follicular cell-derived non-anaplastic thyroid carcinoma instead of RAI-R differentiated thyroid carcinoma to more accurately reflect the spectrum of patients that may require systemic therapy.
   1. Strongly agree
   2. Agree
   3. Neither agree nor disagree
   4. Disagree
   5. Strongly disagree

If disagree/strongly disagree, what would you recommend instead: [open response]

*Histopathology, Molecular Features and Risk Stratification*

1. Poorly differentiated thyroid carcinoma can be best defined by the presence of the following histopathologic, molecular and clinical features: [select multiple]
2. Intermediate morphologic features between follicular cell-derived differentiated and anaplastic thyroid carcinomas
3. Thyroglobulin-producing
4. Intermediate behaviour between well-differentiated and anaplastic carcinoma
5. High mitotic index (≥3 mitoses per 2 mm2)
   1. Presence of tumor necrosis
   2. Not enough evidence to provide a strict recommendation
6. High-grade differentiated thyroid carcinoma can be best defined by the presence of the following histopathologic, molecular and clinical features: [select multiple]
7. A growth pattern similar to well-differentiated tumours
8. Papillary growth pattern in the majority of cases
9. Necrosis and/or excess mitotic activity (5 mitoses/10 hpf)
10. Vascular, lymphatic, perineural and extrathyroidal invasion common
11. Solid trabecular or insular growth
12. How should FDG-PET be used for diagnosis and follow-up of RAI-R follicular cell-derived non-anaplastic thyroid carcinoma
13. For routine pre-op or pre-systemic treatment staging
14. For pre-op or pre-systemic treatment staging of high-risk disease
15. For pre-op or pre-systemic treatment staging of recurrent disease
16. For post-op staging of recurrent disease
17. Other major indications: [open response]
18. What features can be used to reliably identify patients with follicular cell-derived non-anaplastic thyroid carcinoma at high risk of being RAI-R? [select multiple]
19. Poorly differentiated thyroid carcinoma
20. High grade differentiated thyroid carcinomas (e.g. high-grade papillary thyroid carcinoma, high-grade follicular thyroid carcinoma, high-grade oncocytic carcinoma of the thyroid)
21. Distant metastases with or without neck involvement
22. FDG-PET positivity
23. Rising serum thyroglobulin
24. Not enough evidence to provide a recommendation
25. Post-operative RAI is not likely to be effective in patients deemed at high risk of being RAI-R; i.e. primary non-responders (based on features listed under “7” above).
26. Strongly agree
27. Agree
28. Neither agree nor disagree
29. Disagree
30. Strongly disagree

If disagree/strongly disagree, what would you recommend instead: [open response]

*Molecular Testing*

1. Germline genetic testing for disease-causing pathogenic variants (e.g. *PTEN*, *DICER1, SDHx, BRCA1/2 [gBRCA], TP53*) should be considered in the workup of **selected patients** with diagnosed thyroid cancer.
2. Strongly agree
3. Agree
4. Neither agree nor disagree
5. Disagree
6. Strongly disagree

If disagree/strongly disagree, what would you recommend instead: [open response]

1. Select all situations which should trigger reflex tissue molecular testing.

- **Pre-operative**

1. Triggered by the pathologist for patients with adverse features (e.g. angioinvasive, high-grade features, adverse tumor subtypes)
2. Triggered by the surgeon, radiation oncologist or rarely, nuclear medicine physician for patients with distant metastases at diagnosis
3. Triggered by the surgeon for patients with unresectable or borderline resectable disease who might be considered for neoadjuvant therapy

- **Post-operative**

1. Triggered by the radiation oncologist/nuclear medicine physician at the first palliative (i.e. non-adjuvant) RAI treatment

- **Recurrence or progression**

1. Triggered by the radiation oncologist or, rarely, nuclear medicine physician for patients with distant metastases at progression
2. Triggered by the medical oncologist (if not yet completed) when a patient is deemed inoperable
3. Triggered by the surgeon when a patient is deemed borderline inoperable or completely inoperable

What other situations should trigger reflex testing and who should be responsible [open response]?

1. Patients should be referred for tumor tissue (somatic) molecular testing based on the need for molecular testing rather than indication for systemic treatment.
2. Strongly agree
3. Agree
4. Neither agree nor disagree
5. Disagree
6. Strongly disagree

If disagree/strongly disagree, what would you recommend instead: [open response]

1. Which of the following tumour sampling options are acceptable for molecular testing? [select multiple]
2. FNA of the primary thyroid tumour
3. Core biopsy of the primary tumour
4. Incisional/excisional biopsy of primary tumour at or metastasis

Provide any rationale for your choice(s) as needed:

1. Categorize the following biomarkers as essential or desirable. For each biomarker include the preferred assay method (i.e. DNA NGS, RNA NGS, IHC, PCR, FISH, etc.).
2. BRAF p.V600E-mutation specific immunohistochemistry
3. BRAF-MOLECULAR
4. *NRAS*
5. *HRAS*
6. *KRAS*
7. *ALK* fusion
8. *RET* fusion
9. *PPARG* fusion
10. *NTRK1* fusion
11. *NTRK2* fusion
12. *NTRK3* fusion
13. Pan-TRK immunohistochemistry
14. ALK fusion specific immunohistochemistry
15. *NUTM1*
16. PTEN immunohistochemistry
17. SDHB immunohistochemistry
18. Pan-RAS Q61R mutation specific immunohistochemistry
19. 5-hmC immunohistochemistry
20. *TERT* promoter alterations

**Therapeutic**

*Treatment – Surgery*

1. Active surveillance is recommended in patients with small volume neck disease in a previously operated field.
2. Strongly agree
3. Agree
4. Neither agree nor disagree
5. Disagree
6. Strongly disagree

If disagree/strongly disagree, what would you recommend instead: [open response]

1. Alternate treatments such as ethanol or radiofrequency ablation should be considered in patients with growing cervical metastatic disease in previously operated fields, safely away from critical structures.
2. Strongly agree
3. Agree
4. Neither agree nor disagree
5. Disagree
6. Strongly disagree

If disagree/strongly disagree, what would you recommend instead: [open response]

1. Rate your agreement with the following statements (1=Strongly disagree, 5=Strongly agree):
2. Resection should be considered in patients with oligometastasis.
3. Resection should be considered in patients where it would not severely worsen the patient’s quality of life.
4. Resection should be considered in patients with newly discovered metastatic disease in the neck in area without previous operation.
5. Resection should be considered in patients with growing recurrent cervical disease confined to a single region or close to major structure with impending invasion.

If disagree/strongly disagree, what would you recommend instead: [open response]

1. Borderline resectable thyroid cancer should be defined as usually large volume cervical disease which would preclude likely R0 resection including invasion into critical structures such as larynx, major vascular structures, or large segment of trachea.
2. Strongly agree
3. Agree
4. Neither agree nor disagree
5. Disagree
6. Strongly disagree

If disagree/strongly disagree, what would you recommend instead: [open response]

1. When deciding about management of airway in patients with locally advanced and/or progressive disease, prior to institution of systemic TKi:
2. Tracheostomy should be inserted as early as possible when risks of disease progression in tracheoesophageal region are of concern.
3. Tracheostomy should be reserved for after systemic therapy has been considered.
4. Tracheostomy should be reserved until after EBRT has been considered.
5. Patient quality of life and end of life wishes should be considered before tracheostomy.

*Treatment – Radiation Medicine*

1. EBRT should be considered in patients who have unresectable gross residual disease, very high risk of recurrence in neck despite all gross disease resected, metastatic disease where surgery is not recommended or desired or post-metastasectomy if still at risk for recurrence (e.g. Brain mets resection, spine met resection).
2. Strongly agree
3. Agree
4. Neither agree nor disagree
5. Disagree
6. Strongly disagree

If disagree/strongly disagree, what would you recommend instead: [open response]

1. Rate your agreement with the following statements (1=Strongly disagree, 5=Strongly agree):
2. SRS/SRT should be offered to eligible patients with CNS metastases
3. SABR for extra-cranial metastases should be considered for selected patients with <5 oligometastases.

If disagree/strongly disagree, what would you recommend instead: [open response]

*Treatment – Systemic Therapy*

1. Neoadjuvant TKi is an appropriate treatment consideration for those with unresectable, locally advanced cancer who may not have received RAI.
2. Strongly agree
3. Agree
4. Neither agree nor disagree
5. Disagree
6. Strongly disagree

If disagree/strongly disagree, what would you recommend instead: [open response]

1. Rate your agreement with the following statements (1=Strongly disagree, 5=Strongly agree):
2. Multikinase inhibitor treatment should not be delayed due to perceived risk of complications including tracheal fistulization
3. TKi should be used first line before tracheostomy

If disagree/strongly disagree, what would you recommend instead: [open response]

1. If there is an actionable mutation, should targeted therapy be used as 1L instead of lenvatinib?
2. *RET* fusion
   - 1. Yes
     2. No
     3. Not enough evidence to conclude
3. *NTRK* fusion
   - 1. Yes
     2. No
     3. Not enough evidence to conclude
4. *BRAF* p.V600E mutation
   - 1. Yes
     2. No
     3. Not enough evidence to conclude
5. Immunotherapy could be considered for 1L treatment in patients who are MSI-high or have high mutational burden carcinomas, in the absence of other actionable mutations.
6. Strongly agree
7. Agree
8. Neither agree nor disagree
9. Disagree
10. Strongly disagree

If disagree/strongly disagree, what would you recommend instead: [open response]

1. Although evidence is limited, patients who develop TKi resistance (with no actionable mutations or who have exhausted targeted options) should be considered for chemotherapy.
2. Strongly agree
3. Agree
4. Neither agree nor disagree
5. Disagree
6. Strongly disagree

If disagree/strongly disagree, what would you recommend instead: [open response]

1. PARPi should be considered in *gBRCA^mut^* patients.
2. Strongly agree
3. Agree
4. Neither agree nor disagree
5. Disagree
6. Strongly disagree

If agree/strongly agree, where would you use PARPi in the current sequence of available treatments?

If disagree/strongly disagree, what would you recommend instead: [open response]

1. Radioactive iodine resensitization therapy should only be considered as part of a clinical trial.
2. Strongly agree
3. Agree
4. Neither agree nor disagree
5. Disagree
6. Strongly disagree

If disagree/strongly disagree, what would you recommend instead: [open response]

**Logistics/Implementation**

*Referral*

1. The following types of patients should be considered for referral to a medical oncologist or endocrine oncologist (select all that apply):
2. Patients who are at high risk of having RAI-R follicular cell-derived non-anaplastic thyroid carcinoma (as defined under question 4 above)
3. Patients who have incurable, locally advanced and/or metastatic disease with evidence of structural disease
4. Patients with structural progression despite prior RAI therapy
5. Patients with short disease-free interval (<12 months) following RAI
6. Patients with FDG-PET positivity having received RAI
7. Which practitioner should be most responsible for identification and referral of patients with potential RAI-R follicular-cell derived non-anaplastic thyroid carcinoma in your region?
8. ENT/Otolaryngology surgeon
9. Head and neck surgeon
10. Primary care clinician (physician/nurse practitioner)
11. General endocrinologist
12. Medical oncologist
13. Nuclear Medicine
14. Radiation oncologist
15. What is the average wait time **from consultation to treatment** in your region for new patients with RAI-R thyroid carcinoma with non-rapidly growing mass(es): [open response]

Which patient types should be expedited for quicker referral: [open response]

*Advocacy*

1. This consensus publication authors should engage the following thyroid cancer support groups, endocrinology, rare disease organizations in Canada for patient advocacy: [open responses]
2. Canadian clinicians should advocate for universal access to tissue molecular testing in patients with follicular cell-derived non-anaplastic thyroid carcinoma.
3. Strongly agree
4. Agree
5. Neither agree nor disagree
6. Disagree
7. Strongly disagree

If disagree/strongly disagree, what would you recommend instead: [open response]

**PubMed Search Strategy**

1. General Thyroid Carcinoma

| Search | Query | Items found |
| --- | --- | --- |
| #1 | "Thyroid Neoplasms"[Mesh] OR "Thyroid cancer, follicular" [Supplementary Concept] OR “thyroid cancer*”[tiab] OR “thyroid neoplasm*”[tiab] OR “thyroid carcinoma*”[tiab] OR “thyroid tumor*”[tiab] OR “thyroid tumour*”[tiab] | 76,298 |
| #2 | “differentiated thyroid cancer*”[tiab] OR DTC[tiab] OR “differentiated thyroid carcinoma*”[tiab] OR “differentiated thyroid neoplasm*”[tiab] OR “differentiated thyroid tumour*”[tiab] OR “differentiated thyroid tumor*”[tiab] | 12,217 |
| #3 | “radioactive iodine”[tiab] OR radioiodine[tiab] OR RAI[tiab] | 2739 |
| #4 | #1 AND #2 AND #3 with Filters: Case Reports, Clinical Study, Clinical Trial, Clinical Trial, Phase I, Clinical Trial, Phase II, Clinical Trial, Phase III, Clinical Trial, Phase IV, Comparative Study, Controlled Clinical Trial, Evaluation Study, Meta-Analysis, Multicenter Study, Observational Study, Practice Guideline, Preprint, Randomized Controlled Trial, Systematic Review, Validation Study, Humans, English | 781 |

Reviewed article titles and abstracts manually for relevance to topic. Eliminated those related to medullary and anaplastic thyroid carcinoma.

1. Defining Poorly Differentiated Thyroid Carcinoma

| Search | Query | Items found |
| --- | --- | --- |
| #1 | “poorly differentiated thyroid cancer*”[tiab] OR “poorly differentiated thyroid carcinoma*”[tiab] | 564 |
| #2 | ident*[tiab] OR defin*[tiab] OR classif*[tiab] OR diagnos*[tiab] | 8,352,684 |
| #3 | #1 AND #2 | 332 |
| #10 | Search: #1 AND #2 With Filters: Case Reports, Clinical Study, Clinical Trial, Clinical Trial, Phase I, Clinical Trial, Phase II, Clinical Trial, Phase III, Clinical Trial, Phase IV, Comparative Study, Controlled Clinical Trial, Evaluation Study, Meta-Analysis, Multicenter Study, Observational Study, Practice Guideline, Preprint, Randomized Controlled Trial, Systematic Review, Validation Study, Humans, English | 59 |

Reviewed articles for relevance and eliminated duplicates with previous searches.

1. Defining High Grade Differentiated Thyroid Carcinoma

| Search | Query | Items found |
| --- | --- | --- |
| #1 | “high-grade”[tiab] OR “high grade”[tiab] With Filters: Case Reports, Clinical Study, Clinical Trial, Clinical Trial, Phase I, Clinical Trial, Phase II, Clinical Trial, Phase III, Clinical Trial, Phase IV, Comparative Study, Controlled Clinical Trial, Evaluation Study, Meta-Analysis, Multicenter Study, Observational Study, Practice Guideline, Preprint, Randomized Controlled Trial, Systematic Review, Validation Study, Humans, English | 17, 540 |
| #2 | “thyroid carcinoma*”[tiab] OR “differentiated thyroid carcinoma*”[tiab] OR “differentiated thyroid cancer*” With Filters: Case Reports, Clinical Study, Clinical Trial, Clinical Trial, Phase I, Clinical Trial, Phase II, Clinical Trial, Phase III, Clinical Trial, Phase IV, Comparative Study, Controlled Clinical Trial, Evaluation Study, Meta-Analysis, Multicenter Study, Observational Study, Practice Guideline, Preprint, Randomized Controlled Trial, Systematic Review, Validation Study, Humans, English | 7, 088 |
| #3 | ident*[tiab] OR defin*[tiab] OR classif*[tiab] OR diagnos*[tiab] With Filters: Case Reports, Clinical Study, Clinical Trial, Clinical Trial, Phase I, Clinical Trial, Phase II, Clinical Trial, Phase III, Clinical Trial, Phase IV, Comparative Study, Controlled Clinical Trial, Evaluation Study, Meta-Analysis, Multicenter Study, Observational Study, Practice Guideline, Preprint, Randomized Controlled Trial, Systematic Review, Validation Study, Humans, English | 1,368,502 |
| #4 | #1 AND #2 AND #3 | 29 |

Reviewed articles for relevance and eliminated duplicates with previous searches.

1. Radioiodine Refractory Risk

| Search | Query | Items found |
| --- | --- | --- |
| #1 | “thyroid carcinoma*”[tiab] OR “differentiated thyroid carcinoma*”[tiab] OR “differentiated thyroid cancer*”[tiab] With Filters: Case Reports, Clinical Study, Clinical Trial, Clinical Trial, Phase I, Clinical Trial, Phase II, Clinical Trial, Phase III, Clinical Trial, Phase IV, Comparative Study, Controlled Clinical Trial, Evaluation Study, Meta-Analysis, Multicenter Study, Observational Study, Practice Guideline, Preprint, Randomized Controlled Trial, Systematic Review, Validation Study, Humans, English | 7,088 |
| #2 | “radioactive iodine-resistan*”[tiab] OR “radioactive iodine-refractory”[tiab] OR “radioactive iodine resistan*”[tiab] OR “radioactive iodine refractory”[tiab] OR “radioiodine-resistant”[tiab] OR “radioiodine-refractory”[tiab] OR “radioiodine resistan*”[tiab] OR “radioiodine refractory”[tiab] OR “RAIR”[tiab] OR “RAI-R”[tiab] With Filters: Case Reports, Clinical Study, Clinical Trial, Clinical Trial, Phase I, Clinical Trial, Phase II, Clinical Trial, Phase III, Clinical Trial, Phase IV, Comparative Study, Controlled Clinical Trial, Evaluation Study, Meta-Analysis, Multicenter Study, Observational Study, Practice Guideline, Preprint, Randomized Controlled Trial, Systematic Review, Validation Study, Humans, English | 176 |
| #3 | risk*[tiab] OR likely[tiab] With Filters: Case Reports, Clinical Study, Clinical Trial, Clinical Trial, Phase I, Clinical Trial, Phase II, Clinical Trial, Phase III, Clinical Trial, Phase IV, Comparative Study, Controlled Clinical Trial, Evaluation Study, Meta-Analysis, Multicenter Study, Observational Study, Practice Guideline, Preprint, Randomized Controlled Trial, Systematic Review, Validation Study, Humans, English | 679,784 |
| #4 | #1 AND #2 AND #3 | 19 |

Reviewed articles for relevance and eliminated duplicates with previous searches.

1. FDG-PET

| Search | Query | Items found |
| --- | --- | --- |
| #1 | “thyroid carcinoma*”[tiab] OR “differentiated thyroid carcinoma*”[tiab] OR “differentiated thyroid cancer*”[tiab] With Filters: Case Reports, Clinical Study, Clinical Trial, Clinical Trial, Phase I, Clinical Trial, Phase II, Clinical Trial, Phase III, Clinical Trial, Phase IV, Comparative Study, Controlled Clinical Trial, Evaluation Study, Meta-Analysis, Multicenter Study, Observational Study, Practice Guideline, Preprint, Randomized Controlled Trial, Systematic Review, Validation Study, Humans, English | 7,088 |
| #2 | “radioactive iodine-resistan*”[tiab] OR “radioactive iodine-refractory”[tiab] OR “radioactive iodine resistan*”[tiab] OR “radioactive iodine refractory”[tiab] OR “radioiodine-resistant”[tiab] OR “radioiodine-refractory”[tiab] OR “radioiodine resistan*”[tiab] OR “radioiodine refractory”[tiab] OR “RAIR”[tiab] OR “RAI-R”[tiab] With Filters: Case Reports, Clinical Study, Clinical Trial, Clinical Trial, Phase I, Clinical Trial, Phase II, Clinical Trial, Phase III, Clinical Trial, Phase IV, Comparative Study, Controlled Clinical Trial, Evaluation Study, Meta-Analysis, Multicenter Study, Observational Study, Practice Guideline, Preprint, Randomized Controlled Trial, Systematic Review, Validation Study, Humans, English | 176 |
| #3 | “FDG-PET”[tiab] OR “FDG PET”[tiab] With Filters: Case Reports, Clinical Study, Clinical Trial, Clinical Trial, Phase I, Clinical Trial, Phase II, Clinical Trial, Phase III, Clinical Trial, Phase IV, Comparative Study, Controlled Clinical Trial, Evaluation Study, Meta-Analysis, Multicenter Study, Observational Study, Practice Guideline, Preprint, Randomized Controlled Trial, Systematic Review, Validation Study, Humans, English | 11, 264 |
| #4 | #1 AND #2 AND #3 | 11 |

Reviewed articles for relevance and eliminated duplicates with previous searches.

1. Germline Genetic Testing

| Search | Query | Items found |
| --- | --- | --- |
| #1 | “thyroid carcinoma*”[tiab] OR “thyroid cancer*”[tiab] OR “differentiated thyroid carcinoma*”[tiab] OR “differentiated thyroid cancer*”[tiab] With Filters: Case Reports, Clinical Study, Clinical Trial, Clinical Trial, Phase I, Clinical Trial, Phase II, Clinical Trial, Phase III, Clinical Trial, Phase IV, Comparative Study, Controlled Clinical Trial, Evaluation Study, Meta-Analysis, Multicenter Study, Observational Study, Practice Guideline, Preprint, Randomized Controlled Trial, Systematic Review, Validation Study, Humans, English | 9,890 |
| #2 | germline[tiab] OR “hereditary cancer*”[tiab] OR “inherited cancer*”[tiab] With Filters: Case Reports, Clinical Study, Clinical Trial, Clinical Trial, Phase I, Clinical Trial, Phase II, Clinical Trial, Phase III, Clinical Trial, Phase IV, Comparative Study, Controlled Clinical Trial, Evaluation Study, Meta-Analysis, Multicenter Study, Observational Study, Practice Guideline, Preprint, Randomized Controlled Trial, Systematic Review, Validation Study, Humans, English | 6,462 |
| #3 | test*[tiab] OR “gene* test*”[tiab] OR screen*[tiab] With Filters: Case Reports, Clinical Study, Clinical Trial, Clinical Trial, Phase I, Clinical Trial, Phase II, Clinical Trial, Phase III, Clinical Trial, Phase IV, Comparative Study, Controlled Clinical Trial, Evaluation Study, Meta-Analysis, Multicenter Study, Observational Study, Practice Guideline, Preprint, Randomized Controlled Trial, Systematic Review, Validation Study, Humans, English | 719,867 |
| #4 | #1 AND #2 AND #3 | 111 |

Reviewed articles for relevance and eliminated duplicates with previous searches.

1. Molecular Testing

| Search | Query | Items found |
| --- | --- | --- |
| #1 | “thyroid carcinoma*”[tiab] OR “thyroid cancer*”[tiab] OR “differentiated thyroid carcinoma*”[tiab] OR “differentiated thyroid cancer*”[tiab] With Filters: Case Reports, Clinical Study, Clinical Trial, Clinical Trial, Phase I, Clinical Trial, Phase II, Clinical Trial, Phase III, Clinical Trial, Phase IV, Comparative Study, Controlled Clinical Trial, Evaluation Study, Meta-Analysis, Multicenter Study, Observational Study, Practice Guideline, Preprint, Randomized Controlled Trial, Systematic Review, Validation Study, Humans, English | 9,891 |
| #2 | “molecular test*”[tiab] OR “somatic test*”[tiab] OR “somatic genetic test*”[tiab] OR “tumor test*”[tiab] OR “tumor genetic test*”[tiab] OR “tumour test*”[tiab] OR “tumour genetic test*”[tiab] With Filters: Case Reports, Clinical Study, Clinical Trial, Clinical Trial, Phase I, Clinical Trial, Phase II, Clinical Trial, Phase III, Clinical Trial, Phase IV, Comparative Study, Controlled Clinical Trial, Evaluation Study, Meta-Analysis, Multicenter Study, Observational Study, Practice Guideline, Preprint, Randomized Controlled Trial, Systematic Review, Validation Study, Humans, English | 2,114 |
| #4 | #1 AND #2 | 68 |

Reviewed articles for relevance and eliminated duplicates with previous searches.

1. Molecular Testing – Sampling

| Search | Query | Items found |
| --- | --- | --- |
| #1 | “thyroid carcinoma*”[tiab] OR “thyroid cancer*”[tiab] OR “differentiated thyroid carcinoma*”[tiab] OR “differentiated thyroid cancer*”[tiab] NOT “medullary thyroid carcinoma*”[tiab] NOT “anaplastic thyroid carcinoma*”[tiab] NOT “indeterminate”[tiab] With Filters: Case Reports, Clinical Study, Clinical Trial, Clinical Trial, Phase I, Clinical Trial, Phase II, Clinical Trial, Phase III, Clinical Trial, Phase IV, Comparative Study, Controlled Clinical Trial, Evaluation Study, Meta-Analysis, Multicenter Study, Observational Study, Practice Guideline, Preprint, Randomized Controlled Trial, Systematic Review, Validation Study, Humans, English | 8,195 |
| #2 | “molecular test*”[tiab] OR “somatic test*”[tiab] OR “somatic genetic test*”[tiab] OR “tumor test*”[tiab] OR “tumor genetic test*”[tiab] OR “tumour test*”[tiab] OR “tumour genetic test*”[tiab] OR “biomarker test*”[tiab] With Filters: Case Reports, Clinical Study, Clinical Trial, Clinical Trial, Phase I, Clinical Trial, Phase II, Clinical Trial, Phase III, Clinical Trial, Phase IV, Comparative Study, Controlled Clinical Trial, Evaluation Study, Meta-Analysis, Multicenter Study, Observational Study, Practice Guideline, Preprint, Randomized Controlled Trial, Systematic Review, Validation Study, Humans, English | 2,339 |
| #3 | sample[tiab] OR sampling[tiab] OR biopsy[tiab] OR FNA[tiab] OR incisional[tiab] OR excisional[tiab] With Filters: Case Reports, Clinical Study, Clinical Trial, Clinical Trial, Phase I, Clinical Trial, Phase II, Clinical Trial, Phase III, Clinical Trial, Phase IV, Comparative Study, Controlled Clinical Trial, Evaluation Study, Meta-Analysis, Multicenter Study, Observational Study, Practice Guideline, Preprint, Randomized Controlled Trial, Systematic Review, Validation Study, Humans, English | 304, 167 |
| #4 | #1 AND #2 AND #3 | 16 |

Reviewed articles for relevance and eliminated duplicates with previous searches.

1. Active Surveillance

| Search | Query | Items found |
| --- | --- | --- |
| #1 | “thyroid carcinoma*”[tiab] OR “thyroid cancer*”[tiab] OR “differentiated thyroid carcinoma*”[tiab] OR “differentiated thyroid cancer*”[tiab] NOT “medullary thyroid carcinoma*”[tiab] NOT “anaplastic thyroid carcinoma*”[tiab] NOT “low risk”[tiab] NOT “low-risk”[tiab] AND Filters: Case Reports, Clinical Study, Clinical Trial, Clinical Trial, Phase I, Clinical Trial, Phase II, Clinical Trial, Phase III, Clinical Trial, Phase IV, Comparative Study, Controlled Clinical Trial, Evaluation Study, Meta-Analysis, Multicenter Study, Observational Study, Practice Guideline, Preprint, Randomized Controlled Trial, Systematic Review, Validation Study, Humans, English | 8,135 |
| #2 | “active surveillance”[tiab] OR “watchful waiting”[tiab] OR “watch and wait”[tiab] OR “watch-and-wait”[tiab] AND Filters: Case Reports, Clinical Study, Clinical Trial, Clinical Trial, Phase I, Clinical Trial, Phase II, Clinical Trial, Phase III, Clinical Trial, Phase IV, Comparative Study, Controlled Clinical Trial, Evaluation Study, Meta-Analysis, Multicenter Study, Observational Study, Practice Guideline, Preprint, Randomized Controlled Trial, Systematic Review, Validation Study, Humans, English | 2,848 |
| #3 | #1 AND #2 | 29 |

Reviewed articles for relevance and eliminated duplicates with previous searches.

1. Resection

| Search | Query | Items found |
| --- | --- | --- |
| #1 | “thyroid carcinoma*”[tiab] OR “thyroid cancer*”[tiab] OR “differentiated thyroid carcinoma*”[tiab] OR “differentiated thyroid cancer*”[tiab] NOT “medullary thyroid carcinoma*”[tiab] NOT “anaplastic thyroid carcinoma*”[tiab] AND Filters: Case Reports, Clinical Study, Clinical Trial, Clinical Trial, Phase I, Clinical Trial, Phase II, Clinical Trial, Phase III, Clinical Trial, Phase IV, Comparative Study, Controlled Clinical Trial, Evaluation Study, Meta-Analysis, Multicenter Study, Observational Study, Practice Guideline, Preprint, Randomized Controlled Trial, Systematic Review, Validation Study, Humans, English | 8,370 |
| #2 | resection[tiab] OR surg*[tiab] AND Filters: Case Reports, Clinical Study, Clinical Trial, Clinical Trial, Phase I, Clinical Trial, Phase II, Clinical Trial, Phase III, Clinical Trial, Phase IV, Comparative Study, Controlled Clinical Trial, Evaluation Study, Meta-Analysis, Multicenter Study, Observational Study, Practice Guideline, Preprint, Randomized Controlled Trial, Systematic Review, Validation Study, Humans, English | 591, 111 |
| #3 | recurr*[tiab] OR metast*[tiab] OR advanced[tiab] AND Filters: Case Reports, Clinical Study, Clinical Trial, Clinical Trial, Phase I, Clinical Trial, Phase II, Clinical Trial, Phase III, Clinical Trial, Phase IV, Comparative Study, Controlled Clinical Trial, Evaluation Study, Meta-Analysis, Multicenter Study, Observational Study, Practice Guideline, Preprint, Randomized Controlled Trial, Systematic Review, Validation Study, Humans, English | 379, 192 |
| #4 | #1 AND #2 AND #3 | 1,535 |

Reviewed articles for relevance and eliminated duplicates with previous searches.

1. Defining Borderline Resectable Disease

| Search | Query | Items found |
| --- | --- | --- |
| #1 | “thyroid carcinoma*”[tiab] OR “thyroid cancer*”[tiab] OR “differentiated thyroid carcinoma*”[tiab] OR “differentiated thyroid cancer*”[tiab] NOT “medullary thyroid carcinoma*”[tiab] NOT “anaplastic thyroid carcinoma*”[tiab] AND Filters: Case Reports, Clinical Study, Clinical Trial, Clinical Trial, Phase I, Clinical Trial, Phase II, Clinical Trial, Phase III, Clinical Trial, Phase IV, Comparative Study, Controlled Clinical Trial, Evaluation Study, Meta-Analysis, Multicenter Study, Observational Study, Practice Guideline, Preprint, Randomized Controlled Trial, Systematic Review, Validation Study, Humans, English | 8,370 |
| #2 | borderline[tiab] OR “border-line”[tiab] OR “border line”[tiab] OR “borderline resectable”[tiab] OR “borderline-resectable”[tiab] OR “border-line resectable”[tiab] OR “border line resectable”[tiab] AND Filters: Case Reports, Clinical Study, Clinical Trial, Clinical Trial, Phase I, Clinical Trial, Phase II, Clinical Trial, Phase III, Clinical Trial, Phase IV, Comparative Study, Controlled Clinical Trial, Evaluation Study, Meta-Analysis, Multicenter Study, Observational Study, Practice Guideline, Preprint, Randomized Controlled Trial, Systematic Review, Validation Study, Humans, English | 12,091 |
| #3 | ident*[tiab] OR defin*[tiab] OR classif*[tiab] OR diagnos*[tiab] AND Filters: Case Reports, Clinical Study, Clinical Trial, Clinical Trial, Phase I, Clinical Trial, Phase II, Clinical Trial, Phase III, Clinical Trial, Phase IV, Comparative Study, Controlled Clinical Trial, Evaluation Study, Meta-Analysis, Multicenter Study, Observational Study, Practice Guideline, Preprint, Randomized Controlled Trial, Systematic Review, Validation Study, Humans, English | 1,368,550 |
| #4 | #1 AND #2 AND #3 | 23 |

Reviewed articles for relevance and eliminated duplicates with previous searches.

1. Ethanol/Radiofrequency Ablation

| Search | Query | Items found |
| --- | --- | --- |
| #1 | “thyroid carcinoma*”[tiab] OR “thyroid cancer*”[tiab] OR “differentiated thyroid carcinoma*”[tiab] OR “differentiated thyroid cancer*”[tiab] NOT “medullary thyroid carcinoma*”[tiab] NOT “anaplastic thyroid carcinoma*”[tiab] AND Filters: Case Reports, Clinical Study, Clinical Trial, Clinical Trial, Phase I, Clinical Trial, Phase II, Clinical Trial, Phase III, Clinical Trial, Phase IV, Comparative Study, Controlled Clinical Trial, Evaluation Study, Meta-Analysis, Multicenter Study, Observational Study, Practice Guideline, Preprint, Randomized Controlled Trial, Systematic Review, Validation Study, Humans, English | 8,370 |
| #2 | “ethanol ablation”[tiab] OR “radiofrequency ablation”[tiab] AND Filters: Case Reports, Clinical Study, Clinical Trial, Clinical Trial, Phase I, Clinical Trial, Phase II, Clinical Trial, Phase III, Clinical Trial, Phase IV, Comparative Study, Controlled Clinical Trial, Evaluation Study, Meta-Analysis, Multicenter Study, Observational Study, Practice Guideline, Preprint, Randomized Controlled Trial, Systematic Review, Validation Study, Humans, English | 5,491 |
| #3 | #1 AND #2 | 30 |

Reviewed articles for relevance and eliminated duplicates with previous searches.

1. EBRT

| Search | Query | Items found |
| --- | --- | --- |
| #1 | “thyroid carcinoma*”[tiab] OR “thyroid cancer*”[tiab] OR “differentiated thyroid carcinoma*”[tiab] OR “differentiated thyroid cancer*”[tiab] NOT “medullary thyroid carcinoma*”[tiab] NOT “anaplastic thyroid carcinoma*”[tiab]] AND Filters: Case Reports, Clinical Study, Clinical Trial, Clinical Trial, Phase I, Clinical Trial, Phase II, Clinical Trial, Phase III, Clinical Trial, Phase IV, Comparative Study, Controlled Clinical Trial, Evaluation Study, Meta-Analysis, Multicenter Study, Observational Study, Practice Guideline, Preprint, Randomized Controlled Trial, Systematic Review, Validation Study, Humans, English | 8,370 |
| #2 | EBRT[tiab] OR “external-beam radiation therapy”[tiab] OR “external beam radiation therapy”[tiab] OR “external beam radiation”[tiab] OR “external-beam radiation”[tiab] AND Filters: Case Reports, Clinical Study, Clinical Trial, Clinical Trial, Phase I, Clinical Trial, Phase II, Clinical Trial, Phase III, Clinical Trial, Phase IV, Comparative Study, Controlled Clinical Trial, Evaluation Study, Meta-Analysis, Multicenter Study, Observational Study, Practice Guideline, Preprint, Randomized Controlled Trial, Systematic Review, Validation Study, Humans, English | 2,388 |
| #3 | #1 AND #2 | 49 |

Reviewed articles for relevance and eliminated duplicates with previous searches.

1. SRS/SRT

| Search | Query | Items found |
| --- | --- | --- |
| #1 | “thyroid carcinoma*”[tiab] OR “thyroid cancer*”[tiab] OR “differentiated thyroid carcinoma*”[tiab] OR “differentiated thyroid cancer*”[tiab] NOT “medullary thyroid carcinoma*”[tiab] NOT “anaplastic thyroid carcinoma*”[tiab]] AND Filters: Case Reports, Clinical Study, Clinical Trial, Clinical Trial, Phase I, Clinical Trial, Phase II, Clinical Trial, Phase III, Clinical Trial, Phase IV, Comparative Study, Controlled Clinical Trial, Evaluation Study, Meta-Analysis, Multicenter Study, Observational Study, Practice Guideline, Preprint, Randomized Controlled Trial, Systematic Review, Validation Study, Humans, English | 8,370 |
| #2 | “stereotactic radiosurgery”[tiab] OR SRS[tiab] OR “stereotactic radiation therapy”[tiab] OR SRT[tiab] AND Filters: Case Reports, Clinical Study, Clinical Trial, Clinical Trial, Phase I, Clinical Trial, Phase II, Clinical Trial, Phase III, Clinical Trial, Phase IV, Comparative Study, Controlled Clinical Trial, Evaluation Study, Meta-Analysis, Multicenter Study, Observational Study, Practice Guideline, Preprint, Randomized Controlled Trial, Systematic Review, Validation Study, Humans, English | 4769 |
| #3 | #1 AND #2 | 16 |

Reviewed articles for relevance and eliminated duplicates with previous searches.

1. SABR

| Search | Query | Items found |
| --- | --- | --- |
| #1 | “thyroid carcinoma*”[tiab] OR “thyroid cancer*”[tiab] OR “differentiated thyroid carcinoma*”[tiab] OR “differentiated thyroid cancer*”[tiab] NOT “medullary thyroid carcinoma*”[tiab] NOT “anaplastic thyroid carcinoma*”[tiab]] AND Filters: Case Reports, Clinical Study, Clinical Trial, Clinical Trial, Phase I, Clinical Trial, Phase II, Clinical Trial, Phase III, Clinical Trial, Phase IV, Comparative Study, Controlled Clinical Trial, Evaluation Study, Meta-Analysis, Multicenter Study, Observational Study, Practice Guideline, Preprint, Randomized Controlled Trial, Systematic Review, Validation Study, Humans, English | 8,370 |
| #2 | “stereotactic ablative radiotherapy”[tiab] OR SBRT[tiab] OR “stereotactic body radiotherapy”[tiab] OR SABR[tiab] AND Filters: Case Reports, Clinical Study, Clinical Trial, Clinical Trial, Phase I, Clinical Trial, Phase II, Clinical Trial, Phase III, Clinical Trial, Phase IV, Comparative Study, Controlled Clinical Trial, Evaluation Study, Meta-Analysis, Multicenter Study, Observational Study, Practice Guideline, Preprint, Randomized Controlled Trial, Systematic Review, Validation Study, Humans, English | 1,777 |
| #3 | #1 AND #2 | 3 |

Reviewed articles for relevance and eliminated duplicates with previous searches.

1. Neoadjuvant TKi

| Search | Query | Items found |
| --- | --- | --- |
| #1 | “thyroid carcinoma*”[tiab] OR “thyroid cancer*”[tiab] OR “differentiated thyroid carcinoma*”[tiab] OR “differentiated thyroid cancer*”[tiab] NOT “medullary thyroid carcinoma*”[tiab] NOT “anaplastic thyroid carcinoma*”[tiab] AND Filters: Case Reports, Clinical Study, Clinical Trial, Clinical Trial, Phase I, Clinical Trial, Phase II, Clinical Trial, Phase III, Clinical Trial, Phase IV, Comparative Study, Controlled Clinical Trial, Evaluation Study, Meta-Analysis, Multicenter Study, Observational Study, Practice Guideline, Preprint, Randomized Controlled Trial, Systematic Review, Validation Study, Humans, English | 8,370 |
| #2 | neoadjuvant[tiab] AND Filters: Case Reports, Clinical Study, Clinical Trial, Clinical Trial, Phase I, Clinical Trial, Phase II, Clinical Trial, Phase III, Clinical Trial, Phase IV, Comparative Study, Controlled Clinical Trial, Evaluation Study, Meta-Analysis, Multicenter Study, Observational Study, Practice Guideline, Preprint, Randomized Controlled Trial, Systematic Review, Validation Study, Humans, English | 11,962 |
| #3 | TKI[tiab] OR “tyrosine kinase inhibitor*”[tiab] AND Filters: Case Reports, Clinical Study, Clinical Trial, Clinical Trial, Phase I, Clinical Trial, Phase II, Clinical Trial, Phase III, Clinical Trial, Phase IV, Comparative Study, Controlled Clinical Trial, Evaluation Study, Meta-Analysis, Multicenter Study, Observational Study, Practice Guideline, Preprint, Randomized Controlled Trial, Systematic Review, Validation Study, Humans, English | 6,602 |
| #4 | #1 AND #2 AND #3 | 4 |

Reviewed articles for relevance and eliminated duplicates with previous searches.

1. Targeted Therapy

| Search | Query | Items found |
| --- | --- | --- |
| #1 | “thyroid carcinoma*”[tiab] OR “thyroid cancer*”[tiab] OR “differentiated thyroid carcinoma*”[tiab] OR “differentiated thyroid cancer*”[tiab] NOT “medullary thyroid carcinoma*”[tiab] NOT “anaplastic thyroid carcinoma*”[tiab] NOT “indeterminate”[tiab] AND Filters: Case Reports, Clinical Study, Clinical Trial, Clinical Trial, Phase I, Clinical Trial, Phase II, Clinical Trial, Phase III, Clinical Trial, Phase IV, Comparative Study, Controlled Clinical Trial, Evaluation Study, Meta-Analysis, Multicenter Study, Observational Study, Practice Guideline, Preprint, Randomized Controlled Trial, Systematic Review, Validation Study, Humans, English | 8,195 |
| #2 | “targeted therap*”[tiab] OR “targeted treat*”[tiab] OR “precision treat*”[tiab] OR “precision therap*”[tiab] OR vemurafenib[tiab] OR dabrafenib[tiab] OR cobimetinib[tiab] OR trametinib[tiab] OR selpercatinib[tiab] OR pralsetinib[tiab] OR larotrectinib[tiab] OR entrectinib[tiab] AND Filters: Case Reports, Clinical Study, Clinical Trial, Clinical Trial, Phase I, Clinical Trial, Phase II, Clinical Trial, Phase III, Clinical Trial, Phase IV, Comparative Study, Controlled Clinical Trial, Evaluation Study, Meta-Analysis, Multicenter Study, Observational Study, Practice Guideline, Preprint, Randomized Controlled Trial, Systematic Review, Validation Study, Humans, English | 8,398 |
| #3 | “radioactive iodine-resistan*”[tiab] OR “radioactive iodine-refractory”[tiab] OR “radioactive iodine resistan*”[tiab] OR “radioactive iodine refractory”[tiab] OR “radioiodine-resistant”[tiab] OR “radioiodine-refractory”[tiab] OR “radioiodine resistan*”[tiab] OR “radioiodine refractory”[tiab] OR “RAIR”[tiab] OR “RAI-R”[tiab] AND Filters: Case Reports, Clinical Study, Clinical Trial, Clinical Trial, Phase I, Clinical Trial, Phase II, Clinical Trial, Phase III, Clinical Trial, Phase IV, Comparative Study, Controlled Clinical Trial, Evaluation Study, Meta-Analysis, Multicenter Study, Observational Study, Practice Guideline, Preprint, Randomized Controlled Trial, Systematic Review, Validation Study, Humans, English | 176 |
| #4 | #1 AND #2 AND #3 | 26 |

Reviewed articles for relevance and eliminated duplicates with previous searches.

1. Immunotherapy

| Search | Query | Items found |
| --- | --- | --- |
| #1 | “thyroid carcinoma*”[tiab] OR “thyroid cancer*”[tiab] OR “differentiated thyroid carcinoma*”[tiab] OR “differentiated thyroid cancer*”[tiab] AND Filters: Case Reports, Clinical Study, Clinical Trial, Clinical Trial, Phase I, Clinical Trial, Phase II, Clinical Trial, Phase III, Clinical Trial, Phase IV, Comparative Study, Controlled Clinical Trial, Evaluation Study, Meta-Analysis, Multicenter Study, Observational Study, Practice Guideline, Preprint, Randomized Controlled Trial, Systematic Review, Validation Study, Humans, English | 9,891 |
| #2 | immunotherap*[tiab] OR “immune checkpoint inhib*”[tiab] OR “immune checkpoint block*”[tiab] OR “immuno-oncol*”[tiab] OR CTLA[tiab] OR PD-1[tiab] OR PD1[tiab] OR PDL1[tiab] OR PD-L1[tiab] OR ICI[tiab] OR ICB[tiab] IO AND Filters: Case Reports, Clinical Study, Clinical Trial, Clinical Trial, Phase I, Clinical Trial, Phase II, Clinical Trial, Phase III, Clinical Trial, Phase IV, Comparative Study, Controlled Clinical Trial, Evaluation Study, Meta-Analysis, Multicenter Study, Observational Study, Practice Guideline, Preprint, Randomized Controlled Trial, Systematic Review, Validation Study, Humans, English | 21,853 |
| #3 | #1 AND #2 | 62 |

Reviewed articles for relevance and eliminated duplicates with previous searches.

1. Chemotherapy

| Search | Query | Items found |
| --- | --- | --- |
| #1 | “thyroid carcinoma*”[tiab] OR “thyroid cancer*”[tiab] OR “differentiated thyroid carcinoma*”[tiab] OR “differentiated thyroid cancer*”[tiab] AND Filters: Case Reports, Clinical Study, Clinical Trial, Clinical Trial, Phase I, Clinical Trial, Phase II, Clinical Trial, Phase III, Clinical Trial, Phase IV, Comparative Study, Controlled Clinical Trial, Evaluation Study, Meta-Analysis, Multicenter Study, Observational Study, Practice Guideline, Preprint, Randomized Controlled Trial, Systematic Review, Validation Study, Humans, English | 9,891 |
| #2 | chemotherap*[tiab] AND Filters: Case Reports, Clinical Study, Clinical Trial, Clinical Trial, Phase I, Clinical Trial, Phase II, Clinical Trial, Phase III, Clinical Trial, Phase IV, Comparative Study, Controlled Clinical Trial, Evaluation Study, Meta-Analysis, Multicenter Study, Observational Study, Practice Guideline, Preprint, Randomized Controlled Trial, Systematic Review, Validation Study, Humans, English | 114,642 |
| #3 | relapsed[tiab] OR refract*[tiab] OR advanced[tiab] AND Filters: Case Reports, Clinical Study, Clinical Trial, Clinical Trial, Phase I, Clinical Trial, Phase II, Clinical Trial, Phase III, Clinical Trial, Phase IV, Comparative Study, Controlled Clinical Trial, Evaluation Study, Meta-Analysis, Multicenter Study, Observational Study, Practice Guideline, Preprint, Randomized Controlled Trial, Systematic Review, Validation Study, Humans, English | 164,424 |
| #4 | #1 AND #2 | 345 |
| #5 | #1 AND #2 AND #3 | 79 |

Reviewed articles for relevance and eliminated duplicates with previous searches.

1. Resensitization

| Search | Query | Items found |
| --- | --- | --- |
| #1 | “thyroid carcinoma*”[tiab] OR “thyroid cancer*”[tiab] OR “differentiated thyroid carcinoma*”[tiab] OR “differentiated thyroid cancer*”[tiab] NOT “medullary thyroid carcinoma*”[tiab] NOT “anaplastic thyroid carcinoma*”[tiab] AND Filters: Case Reports, Clinical Study, Clinical Trial, Clinical Trial, Phase I, Clinical Trial, Phase II, Clinical Trial, Phase III, Clinical Trial, Phase IV, Comparative Study, Controlled Clinical Trial, Evaluation Study, Meta-Analysis, Multicenter Study, Observational Study, Practice Guideline, Preprint, Randomized Controlled Trial, Systematic Review, Validation Study, Humans, English | 8,370 |
| #2 | resensitiz*[tiab] OR resensitis*[tiab] OR re-sensitiz*[tiab] OR re-sensitis*[tiab] OR redifferent*[tiab] OR re-different*[tiab] AND Filters: Case Reports, Clinical Study, Clinical Trial, Clinical Trial, Phase I, Clinical Trial, Phase II, Clinical Trial, Phase III, Clinical Trial, Phase IV, Comparative Study, Controlled Clinical Trial, Evaluation Study, Meta-Analysis, Multicenter Study, Observational Study, Practice Guideline, Preprint, Randomized Controlled Trial, Systematic Review, Validation Study, Humans, English | 211 |
| #3 | “radioactive iodine-resistan*”[tiab] OR “radioactive iodine-refractory”[tiab] OR “radioactive iodine resistan*”[tiab] OR “radioactive iodine refractory”[tiab] OR “radioiodine-resistant”[tiab] OR “radioiodine-refractory”[tiab] OR “radioiodine resistan*”[tiab] OR “radioiodine refractory”[tiab] OR “RAIR”[tiab] OR “RAI-R”[tiab] AND Filters: Case Reports, Clinical Study, Clinical Trial, Clinical Trial, Phase I, Clinical Trial, Phase II, Clinical Trial, Phase III, Clinical Trial, Phase IV, Comparative Study, Controlled Clinical Trial, Evaluation Study, Meta-Analysis, Multicenter Study, Observational Study, Practice Guideline, Preprint, Randomized Controlled Trial, Systematic Review, Validation Study, Humans, English | 176 |
| #4 | #1 AND #2 AND #3 | 7 |
| #5 | #1 AND #2 | 20 |

Reviewed articles for relevance and eliminated duplicates with previous searches.

# Supplementary Figures and Tables

## Supplementary Tables

**Supplementary Table 1. Summary of Recommendations**

| **#** | **Recommendation** | **Strength of Recommendation** | **Quality of Evidence** |
| --- | --- | --- | --- |
| 3.1 | Follicular cell-derived non-anaplastic thyroid carcinoma at high risk of being RAIR can be identified by the presence of one or more of the following:   - [18F]2-fluoro-2-deoxy-D-glucose-positron emission tomography (FDG-PET) positivity - PDTC - High-grade DTC (e.g., high-grade papillary thyroid carcinoma, high-grade follicular thyroid carcinoma, high-grade oncocytic carcinoma of the thyroid) - DTC with no high-grade features but showing adverse features (which may be histologic and/or molecular adverse [high-risk] features) strongly associated with RAIR disease | Weak | Low |
| 3.2a | The following types of patients **should be referred** to a medical oncologist, endocrine oncologist, or discussed at a multidisciplinary tumor board:   - Patients with structural progression within 12 months of prior RAI therapy   The following types of patients **should be considered for referral** to a medical oncologist, endocrine oncologist, or discussed at a multidisciplinary tumor board:   - Patients who are at high risk of RAIR follicular cell-derived non-anaplastic thyroid carcinoma (as defined above under section 3.1) - Patients who have incurable, locally advanced and/or metastatic disease with evidence of structural disease - Patients with short disease-free interval (<12 months) following RAI - Patients with FDG-PET avid disease - Patients with concerning histopathologic findings (as defined above under section 3.1) | Strong | Insufficient -Expert Opinion |
| 3.2b | The following types of patients should be flagged for expedited referral:   - Patients with rapidly progressing neck masses - Patients who are RAI-naïve or RAIR with symptomatic/rapidly progressing disease (in high-risk population) - Patients whose disease is not amenable to local therapy and/or already deemed inoperable or borderline resectable - Patients with high-grade follicular cell-derived non-anaplastic thyroid carcinoma (including PDTC and high-grade DTC) - Patients with bulky disease and/or of higher stage - Patients with disease at risk of causing morbidity or mortality, including but not limited to impending structural/organ complications | Strong | Insufficient -Expert Opinion |
| 3.2c | The clinician with thyroid cancer expertise who follows patients after RAI treatment should be the most responsible physician for identification and referral of patients with potential RAIRTC. | Strong | Insufficient - Expert Opinion |
| 3.3 | Genetic testing for disease-causing germline (constitutional) pathogenic variants (e.g., phosphatase and tensin homolog [*PTEN]*, *DICER1*, succinate dehydrogenase [*SDHx*], *TP53*) should be considered in the workup of select patients with diagnosed follicular cell-derived thyroid carcinoma, such as those with unique histomorphological and immunohistochemical features that may indicate inherited disease, or patients with hereditary cancer syndromes. | Weak | Low |
| 3.4a | Molecular testing should be performed where clinically relevant and actionable, considering both therapeutic and potential prognostic implications. | Strong | Insufficient - Expert Opinion |
| 3.4b | The following scenarios should trigger molecular testing:   - Pre-operative: Triggered by the pathologist for patients with adverse histologic features (e.g., angioinvasive, high-grade features, morphologic dedifferentiation, adverse tumor subtypes) - Pre-operative: Triggered by the surgeon, radiation oncologist, or nuclear medicine physician for patients with distant metastases at diagnosis - Pre-operative: Triggered by the surgeon for patients with unresectable or borderline resectable disease who might be considered for systemic neoadjuvant therapy - Post-operative: Triggered by the radiation oncologist/nuclear medicine physician at the first palliative (i.e., non-adjuvant) RAI treatment - Recurrence or progression: Triggered by the radiation oncologist or, rarely, nuclear medicine physician for patients with distant metastases at progression - Recurrence or progression: Triggered by the medical oncologist/endocrinologist (if not yet completed) when a patient is deemed inoperable - Recurrence or progression: Triggered by the surgeon when a patient is deemed borderline inoperable or completely inoperable | Strong | Insufficient - Expert Opinion |
| 3.5 | Canadian clinicians should advocate for improved molecular testing, including optimal timing, type of material used, and greater access at tertiary centres, to raise assessment of follicular cell-derived thyroid carcinoma to the level of other solid tumors. | Weak | Insufficient - Expert Opinion |
| 3.6a | The following biomarkers are **essential** to obtain in patients with RAIRTC:   - *BRAF* p.V600E-specific immunohistochemistry - *BRAF* molecular - *RET* fusion - *NTRK* fusions (*NTRK1*, *NTRK2*, *NTRK3*) | Strong | Insufficient - Expert Opinion |
| 3.6b | The following biomarkers are **desirable** to obtain in patients with RAIRTC if possible, considering sample availability and testing accessibility:   - *NRAS* - *HRAS* - *KRAS* - *ALK* fusion - Peroxisome proliferator activated receptor gamma (*PPARG*) fusion - *ALK* fusion-specific immunohistochemistry - *TERT* promoter alterations - NUT midline carcinoma family member 1 (*NUTM1*) - PTEN immunohistochemistry - Succinate dehydrogenase complex iron sulfur subunit B (SDHB) immunohistochemistry - Pan-RAS Q61R mutation-specific immunohistochemistry - 5-hydroxymethylcytosine (5-hmC) immunohistochemistry | Weak | Insufficient - Expert Opinion |
| 3.7a | Active surveillance is recommended in patients with small volume neck disease, especially in a previously operated field. | Weak | Low |
| 3.7b | Resection of recurrent/metastatic disease should be considered in the following scenarios:   - Patients with oligometastases - Patients in whom it would not severely worsen quality of life - Patients with newly discovered metastatic disease in the neck in areas without previous operation - Patients with growing recurrent cervical disease confined to a single region or close to major structure with impending invasion | Weak | Insufficient - Expert Opinion |
| 3.7c | Borderline resectable follicular cell-derived thyroid carcinoma should be defined as large volume cervical disease, which would preclude likely R0 resection due to either bulky and/or widespread lymphadenopathy (e.g., level VII or retropharyngeal) and/or invasion into critical structures such as larynx, major vascular structures, or large segment of trachea. | Weak | Insufficient - Expert Opinion |
| 3.8a | When deciding about airway management in patients with locally advanced and/or progressive unresectable or borderline resectable disease, prior to institution of systemic TKi therapy, patient quality of life and end-of-life wishes should be considered before tracheostomy. | Weak | Insufficient - Expert Opinion |
| 3.8b | TKi may be considered prior to tracheostomy in select patients with careful consideration of risk versus benefit and in discussion with the patient. | Weak | Insufficient - Expert Opinion |
| 3.8c | Multikinase inhibitor treatment should not be delayed in select cases due to perceived risk of complications, including tracheal fistulization. | Weak | Insufficient - Expert Opinion |
| 3.9a | External beam radiotherapy (EBRT) should be considered in patients who have unresectable gross residual disease, very high risk of recurrence in neck despite all gross disease resected, metastatic disease where surgery is not recommended or desired, or post-metastasectomy if risk or morbidity of recurrence remains high (e.g., brain metastases resection, spine metastases resection). | Weak | Low |
| 3.9b | Stereotactic radiosurgery or stereotactic radiotherapy (SRS/SRT) should be offered to eligible patients with central nervous system metastases after appropriate neurosurgical consultation. | Weak | Low |
| 3.9c | Stereotactic ablative radiotherapy (SABR) for extra-cranial metastases should be considered for selected patients with ≤5 oligometastases. | Weak | Low |
| 3.10 | Alternative locoregional treatments such as ethanol or radiofrequency ablation may be considered in patients with growing cervical metastatic disease in previously operated fields, safely away from critical structures. | Weak | Low |
| 3.11 | Neoadjuvant TKi should be considered for those with unresectable and borderline resectable locally advanced thyroid carcinoma who may not have received RAI. | Strong | Low |
| 3.12 | Patients with confirmed, clinically actionable genomic alterations should be considered for targeted therapy, considering individual efficacy/safety needs and access. | Strong | Low |
| 3.13a | In patients for whom other modalities and therapeutics have been exhausted, who do not have actionable targets, and are eligible, immune checkpoint inhibitors could be considered as treatment. | Weak | Low |
| 3.13b | Although evidence is very limited, chemotherapy may be considered in select cases where there are no other therapeutic options, including targeted treatment, immune checkpoint inhibitors, or clinical trials/research protocols. | Weak | Low |
| 3.14 | RAI resensitization therapy should ideally be considered as part of a clinical trial. | Weak | Low |
